# Field‐Validated Detection of *Aureoumbra lagunensis* Brown Tide Blooms in the Indian River Lagoon, Florida, Using Sentinel‐3A OLCI and Ground‐Based Hyperspectral Spectroradiometers

**DOI:** 10.1029/2019GH000238

**Published:** 2020-06-20

**Authors:** Taylor J. Judice, Edith A. Widder, Warren H. Falls, Dulcinea M. Avouris, Dominic J. Cristiano, Joseph D. Ortiz

**Affiliations:** ^1^ Department of Geology Kent State University Kent OH USA; ^2^ Ocean Research and Conservation Association Fort Pierce FL USA

**Keywords:** *Aureoumbra*, brown tide, harmful algal blooms, satellite remote sensing, VPCA spectral decomposition, nutrients

## Abstract

Frequent *Aureoumbra lagunensis* blooms in the Indian River Lagoon (IRL), Florida, have devastated populations of seagrass and marine life and threaten public health. To substantiate a more reliable remote sensing early‐warning system for harmful algal blooms, we apply varimax‐rotated principal component analysis (VPCA) to 12 images spanning ~1.5 years. The method partitions visible‐NIR spectra into independent components related to algae, cyanobacteria, suspended minerals, and pigment degradation products. The components extracted by VPCA are diagnostic for identifiable optical constituents, providing greater specificity in the resulting data products. We show that VPCA components retrieved from Sentinel‐3A Ocean and Land Colour Instrument (OLCI) and a field‐based spectroradiometer are consistent despite vast differences in spatial resolution (~50 cm vs. 300 m). Furthermore, the VPCA components associated with *A*. *lagunensis* in both spectral datasets indicate high correlations to Ochrophyta cell counts (R^2^ **≥** 0.92, *p* < 0.001). Recombining components exhibiting a red‐edge response produces a Chl a algorithm that outperforms empirical band ratio algorithms and preforms as well or better than a variety of semianalytical algorithms. The results from the VPCA spectral decomposition method are more efficient than traditional Empirical Orthogonal Function or PCA, requiring fewer components to explain as much or more variance. Overall, our observations provide excellent validation for Sentinel‐3A OLCI‐based VPCA spectral identification and indicate *A*. *lagunensis* was highly concentrated within the Banana River region of the IRL during the study. These results enable improved brown tide monitoring to identify blooms at an early stage, allowing more time for stakeholder response to this public health problem.

## Key Points

- A novel remote sensing method sees brown tide in Indian River Lagoon, Florida, separate from suspended sediment and cyanobacteria
- Handheld optical instrument and satellite measurements, confirmed by cell counts, and pigment concentrations agree with minimal error
- The water quality harmful algal bloom identification approach can be used with a variety of sensors across a wide range of water bodies

## 1 Introduction

One recurring type of harmful algal boom (HAB) that has grown dominant in portions of the northern Indian River Lagoon (IRL), particularly the Banana River (Barile, 2018; Lapointe et al., 2015, 2017), is the brown tide caused by the pelagophyte *Aureoumbra lagunensis*. This organism, a member of the Phylum Ochrophyta, achieves its name due to the presence of the brown accessory pigment fucoxanthin (http://www.algaebase.org/search/species/detail/?species_id=36722) (Gobler et al., 2013). Although *A*. *lagunensis* is not a toxin producer, it disrupts ecosystems because its extreme bloom concentration blocks sunlight and has led to the extensive die‐off of seagrass, a vital estuarine habitat, with negative economic impacts. Along with *Karenia brevis* (“red tide”), the propagation of *A*. *lagunensis* along the North American coastline has been monitored as it migrated through areas of Texas, Cuba, and Florida (Hall et al., 2018; Koch et al., 2014). The persistence of this HAB‐forming species in coastal waters is due to its resiliency in hypersaline conditions with limited P and low light conditions in shallow, moderately turbid environments (DeYoe et al., 1997; Liu et al., 2001). In many cases, *A*. *lagunensis* is adapted to low P conditions (Kang et al., 2015) and grows well in waters with high N:P ratios.

Field research has linked the potential sources of nutrient pollution causing brown tides to agricultural fertilizer runoff (Zhang et al., 2007), leeching of N from onsite sewage disposal systems (OSDSs, Barile, 2018; Lapointe et al., 2015, 2017), and the accumulation of legacy nutrients resuspended by natural and human‐induced actions (Dunne et al., 2011; Fox & Trefry, 2018; Reddy et al., 2011; Yang et al., 2013). Some research suggests that the limiting nutrient for brown tide is organic nitrogen derived from OSDS, which is ultimately reduced to ammonium or urea (Gobler & Sañudo‐Wilhelmy, 2001; Gobler & Sunda, 2012; Kang et al., 2015; Lapointe et al., 2017). Whether the nutrient pollution is sourced from agricultural or septic runoff, the permeable limestone and sandstone bedrock of Florida produces a soil that has poor retention of N and P, resulting in export of these nutrients to water bodies (McNeal et al., 1995). This results in more frequent HABs during the wet season (Chamberlain & Hayward, 1996). While field sampling research has provided crucial information on the distribution of phytoplankton across the IRL (Phlips et al., 2011), satellite‐based remote sensing of the IRL provides a cost‐effective way to monitor the impact of nutrient pollution over this relatively large body of water that would otherwise be difficult to characterize using traditional field sampling methods alone (Kamerosky et al., 2015).

Remote sensing technology has improved with time, shifting from low‐band count, broad‐band multispectral instruments (e.g. early Landsat multispectral scanners) to more recent high‐band count, multispectral tools (e.g., MERIS and Sentinel‐3A/B Ocean and Land Colour Instrument; OLCI), and early hyperspectral sensors (Hyperion) or the Hyperspectral Imager for Coastal Oceanography (HICO). Those sensors paved the way for planned hyperspectral instruments with hundreds of spectral bands and global coverage (e.g. Plankton, Aerosol, Cloud ocean Ecosystem Ocean Color Imager [PACE OCI], and the Surface Biology and Geology [SBG] mission concept). The addition of specific narrow bands, centered on 620 nm, can also aid in the identification of accessory pigments, such as phycocyanin (Ogashawara & Li, 2019). Although phycocyanin has its peak absorption at 620 nm, its spectral response is broad, extending from ~550 to ~675 nm (Ortiz et al., 2013). While phycocyanin is assumed to be indicative of cyanobacteria in inland waters, it is also present in Cryptophytes and Rhodophytes (Dring, 1982). The increasing number of bands on high‐band count multispectral and hyperspectral sensors particularly when used in optically complex environments result in the inclusion of significant amounts of redundant information because all bands in the visible spectrum are highly correlated (Ortiz et al., 2013, 2017, 2019). New methods of analysis designed to work with redundant information and to capitalize on extraction of spectral shape information are needed.

Various approaches have been developed to characterize HABs through remote sensing of shallow, optically complex waters. These can be grouped into empirical, semianalytical, and analytical methods (Ali et al., 2016). Empirical approaches make use of bands or band ratios (e.g., Han & Rundquist, 1997) that are calibrated or tuned by regression analysis to one or more water quality parameters of interest, such as Chlorophyll a (Chl a), phycocyanin, turbidity, Secchi depth, or colored dissolved organic matter (CDOM) (Kirkpatrick et al., 2011; Slonecker et al., 2016). Semianalytical or analytical methods either make use of theoretical relationships to identify spectral regions of interest (Mishra & Mishra, 2014; Ogashawara & Li, 2019; Simis et al., 2005) or conduct spectral inversion methods (Alcântara et al., 2009; Demetriades‐Shah et al., 1990; Eismann, 2012; Laliberté et al., 2018; Moberg et al., 2002; Mobley et al., 2003; Soja‐Woźniak et al., 2017) to retrieve information related to specific compounds or classes of materials present in the water. Yet, remote sensing of HABs in optically complex environments can be complicated by interference from bottom reflectance or other in‐water constituents including suspended sediment, pigment degradation products (e.g., CDOM), and pigments associated with non‐HAB autotrophs (Kudela et al., 2015; Laliberté et al., 2018). Some approaches attempt to correct for the influence of phycocyanin‐related bands on Chl a and/or for Chl a on phycocyanin‐related bands (Mishra & Mishra, 2014; Ogashawara & Li, 2019; Simis et al., 2005).

Traditional methods have provided several standard metrics for Chl a estimation (Kamerosky et al., 2015; Witter et al., 2009). Kamerosky et al. (2015) applied two Red‐NIR remote sensing algorithms to the IRL to estimate Chl a concentration during an event that has been referred to as the 2011 super bloom. The regressions they employed were able to reconstruct on the order of 40 to 70% of the Chl a variance with errors that exceeded 30 (ug/L), indicating considerable optical interference. The remote sensing challenge in optically complex environments is to untangle the intercorrelated optical signals (Laliberté et al., 2018; Ogashawara & Li, 2019). However, limitations of these standard remote sensing methods arise because many types of phytoplankton contain Chl a, (Sze, 1986) and HABs are often not monospecific, but rather heterogenous in composition (Binding et al., 2019; Hall et al., 2013, 2018; Paerl et al., 2008). There is also evidence that toxic and nontoxic strains of the same HAB‐forming cyanobacteria taxon have different pigment composition (Islam & Beardal, 2017) and spectral signatures (Akins et al., 2020) when grown in culture under identical conditions. To address these issues, we employ the Kent State University (KSU) varimax‐rotated, principal component analysis (VPCA) spectral decomposition method and compare it with traditional methods.

The KSU VPCA method allows HAB spectral constituents mixed in the water column to be detected at lower levels before they dominate the entire reflectance derivative signal. By remotely detecting minor HAB constituents in water using the VPCA spectral decomposition method, remediation efforts can be implemented earlier, providing additional warning in areas such as the IRL. We hypothesize that the VPCA spectral components extracted from the Sentinel‐3A OLCI images that are identified as algal constituents will exhibit spatial patterns that are highly correlated with field measurements of autotrophic pigments and phylum‐level cell counts collected within ±2 days of each other. Because the method can differentiate the spectral signals from independent constituents, this will allow development of improved algorithms for Chl a estimation by separating pigment‐related components from nonpigment‐related components, providing greater specificity. Moreover, given the state of sewage treatment with OSDS in the IRL, we predict that components associated with brown tide algae will be concentrated in the Banana River region, building upon the results of Barile (2018). The results from this study were part of a larger 2‐year‐long research project on the IRL designed to demonstrate the applicability of the method following prior studies conducted in the nutrient‐polluted waters of Lake Erie (Ali et al., 2014, 2016; Ali & Ortiz, 2016; Avouris & Ortiz, 2019; Ortiz et al., 2017, 2019) and the meso‐ to oligotrophic waters of the U.S. Virgin Islands (Schlaerth, 2018).

## 2 Study Site

**Figure 1:**
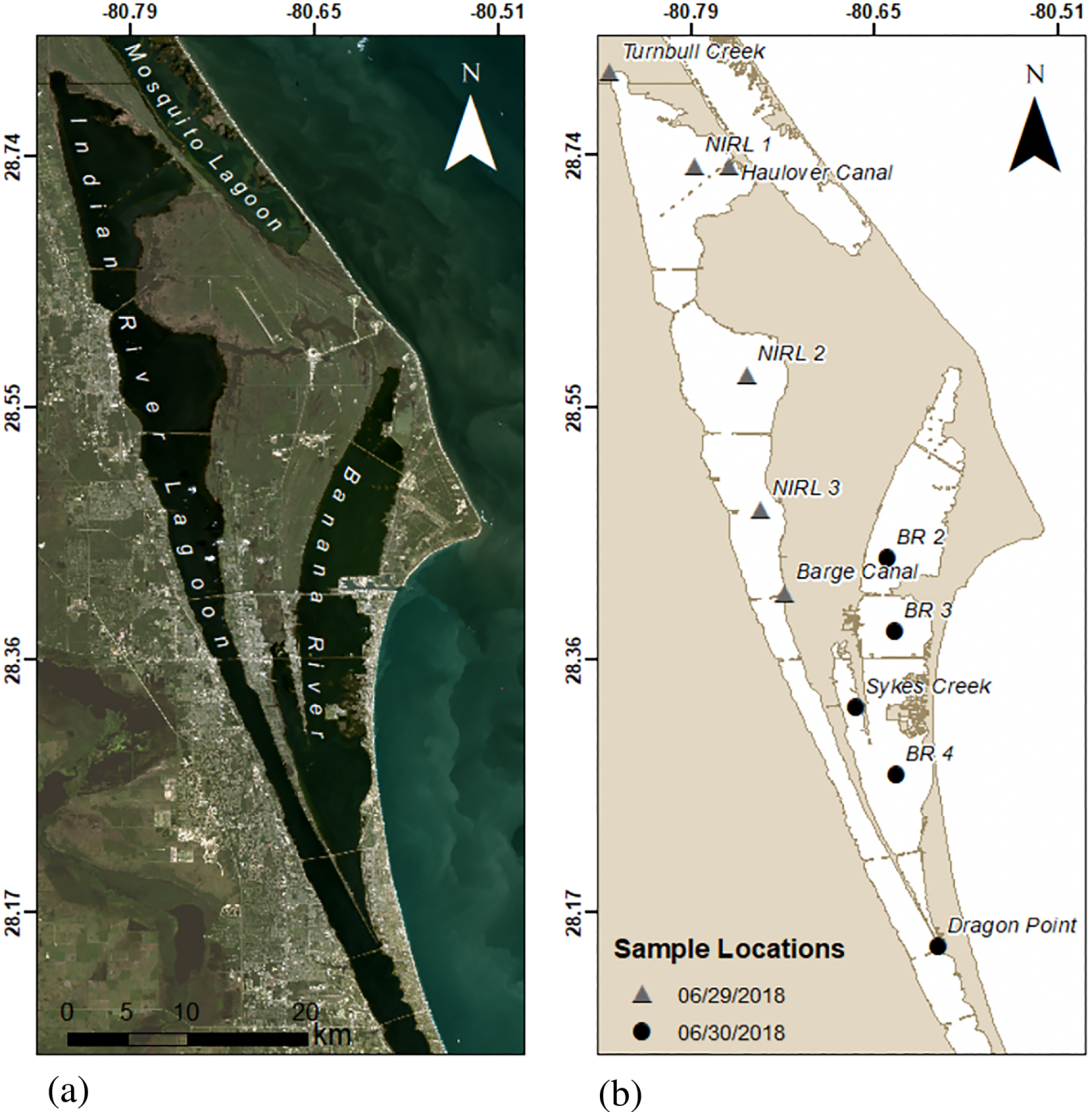
Location map of the northern Indian River lagoon. (a) True color composite image from Landsat‐8 OLI acquired on November 17, 2017. (b) Location of in situ measurements obtained for cell count analysis, hyperspectral Analytical Spectral Devices™ measurements, YSI EXO2 sonde, and Secchi depths on June 29 and 30, 2018. Sites labeled as Turnbull Creek, Haulover Canal, Barge Canal, Sykes Creek, and Dragon Point are locations of piling‐mounted Kilroy instruments for water measurements.

Located along the southeast coast of Florida, the IRL is a shallow‐water estuary of ~5,500 km^2^ area stretching along 250 km of coastline (Figure 1). The IRL encompasses three major lagoons: Mosquito Lagoon in the north, Banana River in the northeast, and the Indian River proper, which extends from Volusia County to Palm Beach County (Figure 1a). In recent years, the growth of HABs within the waters of the IRL have devastated populations of marine life and hindered the local economy (Barile, 2018; Chamberlain & Hayward, 1996; Dybas, 2002; Gobler et al., 2013; Lapointe et al., 2015, 2017; McNeal et al., 1995; Metcalf et al., 2018; Trocine & Trefry, 1996; Yang et al., 2013). The increasing frequency of cyanobacterial and other types of HABs has posed an environmental hazard of growing concern to the more than 1 million residents living along the IRL through fish kills and the negative public health impacts of cyanotoxins, including neurodegenerative and liver diseases (Metcalf et al., 2018). Historically, the IRL has also provided a sanctuary for migratory animals such as dolphins, manatees, and several species of birds. It has been described as one of the most biodiverse regions in the United States (Dybas, 2002). In addition to its importance to wildlife (Sime, 2005), the IRL provides an important economic resource for the local tourism and fishery industries. The compounded environmental issues facing the IRL region have motivated research efforts, combining the resources of field instrumentation and recently developed satellite remote sensing techniques to improve best management practices for the IRL watershed and to reduce nutrient‐rich runoff that favors HAB development.

## 3 Materials and Methods

The full methods section can be found in the supporting information online. An abbreviated version is provided here due to space constraints.

### 3.1 Continuous Autonomous Field Sampling

We conducted remote sensing analysis of Sentinel‐3A OLCI images that covered the northern IRL over an ~1.5‐year time span (7/31/17 to 1/1/19) to isolate the signature of the brown tide that develops there (Table 1). For field comparison with our remote sensing results, we employed several types of measurements, including continuous water‐quality data, obtained using the Ocean Research and Conservation Association's (ORCA) piling‐mounted water‐quality measuring Kilroy™ instrumentation (Thosteson et al., 2009). A total of five fixed stations housed automated Kilroy™ environmental sensing systems that were operated and maintained by ORCA during the course of the study (Figures 1b and S1a,b in the supporting information). These devices record real‐time water‐quality parameters including Chl a and Blue‐Green Algae phycoerythrin (BGA‐PE) as a measure of blue‐green algae continuously in ~30‐min increments year‐round with occasional interruptions for regular maintenance.

**Table 1:** Sentinel‐3A OLCI Image Date and Acquisition Times

Date^a^	Acquisition time (UTC)^b^
8/1/2017	15:10
10/16/2017	15:40
11/17/2017	15:09
3/9/2018	15:06
3/23/2018	15:44
3/24/2018	15:18
6/9/2018	15:21
6/24/2018	15:33
6/28/2018	15:29
8/17/2018	15:33
11/2/2018	15:36
11/21/2018	15:44

a Field operations were conducted on 6/29‐30/2018 to collect samples for image calibration.

^b^ Subtract five hours to convert to local time.

The time series of fluorometrically measured pigments available from the Kilroy™ systems are located at Turnbull Creek, Haulover Canal, Barge Canal, Sykes Creek, and Dragon Point. Sykes Creek is the only Kilroy™ sensor that was located within the Banana River. The Kilroy™ instruments are calibrated against rhodamine dye for each parameter using a two‐point calibration in the lab following manufacturer specifications before deployment. For Chl a, the measurement range is from 0 to 400 ug/L. For the BGA‐PE probe, the measurement range is from 0 to 280 ug/L. A field‐based validation of the Barge Canal Kilroy Chl a and BGA‐PE probes based on 11 months of field check data over a 12‐month period yields *R* = 0.95, R^2^ = 0.91, and RMSE = 12.82 ug/L with *p* = 6.2 × 10^−6^ for Chl a, and *R* = 0.93, R^2^ = 0.87 and RMSE = 23.95 ug/L with *p* = 2.0 × 10^−5^ for BGA‐PE, documenting the stability of the calibrations in the field.

### 3.2 Field Campaign for Remote Sensing Calibration

For image calibration, we implemented field sampling in the IRL on June 29–30, 2018, during a peak in the brown tide. Sampling was conducted from small boats with the assistance of collaborators from ORCA. Measurements were collected from 11 locations in the IRL: six samples from the northern IRL and five samples from the Banana River (Figure 1b; Table S1). The field work was conducted at each of the Kilroy sites and additional locations selected to provide geographic coverage for comparison with the image acquired on June 28, 2018. Nine sites were cloud‐free for comparison with remote sensing pixels. At each station, we measured hyperspectral surface reflectance using a handheld spectroradiometer and hydrographic parameters with a YSI EXO2 Multiparameter sonde (including Chl a and BGA‐PE), and collected water samples for single blind, cell count density (cells/L) and biovolume (**μ**m^3^/L) measurements by an independent, commercial lab (BSA Environmental Services, Inc., Beachwood, OH), along with suspended sediment samples, and Secchi depth measurements. We compare against cell counts because they are the metric employed by water quality managers (e.g., WHO, OEPA, and USACE‐Pittsburgh) to assess HAB conditions and take policy action (Chorus & Bartram, 1999; Davis et al., 2019; OEPA, 2019; USACE‐Pittsburgh, 2020; Wynne et al., 2010). Cell counts also provide algal and cyanobacterial community abundance information useful to assess the reliability of the spectral decomposition method employed here.

During field surveys, a YSI EXO2 Multiparameter sonde was used at all locations to record profiles of Chl a, BGA‐PE, pH, salinity, and turbidity. For cell count analyses, two replicate 125‐mL surface water samples were collected at each of the 11 locations. Once obtained, the cell count samples were preserved using 2 mL of 5% Lugols solution per 25 mL of sample. All water and wet sediment samples were kept cold on ice until returning to the lab for processing and shipment.

Using an Analytical Spectral Devices™ (ASD) FieldSpec® Handheld 2 (HH2) hyperspectral spectroradiometer, we measured the absolute surface water reflectance at the 11 sample locations using a 10° field of view foreoptic attachment, yielding a pixel size of ~50 cm given the elevation of the instrument above the water surface. Measurements were collected to avoid sun glint, but the absolute measurement geometry is less important in our application that traditional remote sensing methods because the KSU spectral decomposition method relies on derivative spectroscopy, rather than direct analysis of reflectance spectra which minimizes geometric interferences (Ortiz et al., 2017). The reflectance spectra from the hyperspectral ASD FieldSpec® HH2 were averaged to 10 nm resolution from 400–700 nm. This provides a more continuous 31 bands compared to the 11 multispectral bands of the Sentinel‐3A OLCI sensor. The hyperspectral data were also resampled to the Sentinel‐3A OLCI band resolution for direct comparison.

The hyperspectral field instrument allows us to obtain surface water reflectance with much less atmospheric interference and much higher sample counts than retrievals from satellite imagery to minimize noise. To minimize noise and increase the signal‐to‐noise ratio (SNR) of the surface water reflectance measurements, each instrument measurement was averaged in several steps. The site‐averaged reflectance values were then transformed to centered‐weighted, first derivatives following Press et al. (1992) to remove the low‐frequency part of the signal, which is dominated by backscatter in preparation for VPCA.

### 3.3 Lab Analysis of Field Data

Cell count samples were kept refrigerated until shipped to BSA Environmental Services, Inc. (Beechwood, Ohio) for analysis. Each sample was identified to the genus and species level, measuring both cell density (cells/L) and cell volume (μm^3^/L). To compare reflectance data with the cell count results, samples were integrated to the Phylum (division) level to account for ambiguity in estimation by remote sensing because the optical signal integrates over the entire plankton community. Results were averaged between the two replicate samples at each location.

To validate the identification for the other spectral components, which were related to minerals, such as clays, hematite, or goethite, SEM‐EDS was used to determine the presence of clays and iron minerals within the dried sediment samples. The sediment collected with the clamshell sampler was wet sieved to grain sizes <63 μm to simulate suspended material that could be observed in the integrated *R*_rs_ spectral signature from Sentinel‐3A OLCI imagery. After sieving, sediment was dried in an oven at 60°C, hand ground with a mortar and pestle to homogenize the signal, and adhered to aluminum tacks for SEM‐EDS analysis. This analysis was aimed at providing a qualitative assessment of presence/absence of clays or iron‐bearing minerals, and thus mineral stoichiometry from the SEM‐EDS was not conducted. The confirmation of clay minerals and iron presence in our field samples provides sufficient validation for the type of mineral constituents identified in the OLCI VPCA decomposition, although there is ambiguity from our SEM measurement as to whether these iron grains were hematite, goethite, or some other iron‐bearing mineral on the basis of the backscatter data alone. In addition, these results are consistent with previous surveys of the IRL, which have found iron in water samples (Trocine & Trefry, 1996).

### 3.4 Lab Culture Reflectance Derivative Spectrum Analysis

In addition to collecting water samples for cell counts, we measured the reflectance of a filtered *A*. *lagunensis* culture provided by ORCA laboratories, as an example spectral response for members of the Ochrophyta. *A*. *lagunensis* was cultured in a 500‐mL flask under standard temperature control and sterile conditions. From the 500 mL of culture, 250 mL of the *A*. *lagunensis* sample was filtered through 47‐mm diameter glass microfiber filters (GF/F) with 0.7 μm pore size at <15 psi to prevent cell breakage (lysing), which minimizes the distortion to pigment packaging that can occur when pigments are extracted by solvents (Bricaud et al., 2004; Lohrenz et al., 2003). These filters accumulate the particulate material within the water but do not capture any CDOM. Following procedures similar to Ortiz et al. (2013), the two filtered water samples were dried at 60°C for ~1 h to remove water. Once the filtered samples dried, they were measured using an ASD FieldSpec® HH2 equipped with a high‐intensity contact probe that utilizes a light source of known optical properties. As with the field reflectance measurements, all GF/F reflectance values were averaged to one reflectance spectra for each site on each day and then transformed to centered derivatives, which removes scattering affects. The spectrum of the culture sample is presented at both 10‐nm hyperspectral resolution and Sentinel‐3A OLCI resolution for comparison with the field collected spectra from the ASD and orbital sensor. The *A*. *lagunensis* spectrum was included in a library of known spectral constituents (Ortiz et al., 2017, 2019), which was used for later component loading identification following the VPCA decomposition.

### 3.5 Remote Sensing Image Analysis and VPCA Spectral Decomposition

The KSU, VPCA method, a Level 3 remote sensing transformation, was developed for decomposing the integrated, spectral signature from optically complex water retrieved by remote sensing instruments. The method partitions reflectance derivative spectra into independent component spectral signatures, which account for quantifiable percentages of the total image variance (Avouris & Ortiz, 2019; Ortiz et al., 2013, 2017, 2019). The method is relatively insensitive to atmospheric error (Ortiz et al., 2017) and can be applied to Level 1 data that are then atmospherically corrected, or to standard Level 2 atmospheric corrected data products. In fact, VPCA improves on traditional atmospheric correction by partitioning signal from noise (Ortiz et al., 2017). In this application, the starting point for the analysis was the standard EUMETSAT Level 2 atmospheric corrected data product for Sentinel‐3A. The atmospheric correction was the ATBD Atmospheric Corrections Bright Water Correction protocol provided by EUMETSAT, which is an updated version of the atmospheric correction algorithm developed for MERIS (Moore & Lavender, 2010; Sentinel‐3 OLCI Marine User Handbook, 2018).

The VPCA method addresses the mixed pixel problem arising from the presence of multiple constituents in optically complex water by partitioning their signals into independent, uncorrelated components (Ortiz et al., 2019). In this way, the method separates signatures of bottom reflection, suspended sediment, and pigment degradation products from various types of algal and cyanobacterial pigments because different processes or targets yield distinct spectral responses (Table S2). The extracted components represent mixtures of constituents that can be characterized by forward, stepwise, principal component regression against a library of known algae, cyanobacteria, pigments, and sediments (Avouris & Ortiz, 2019; Ortiz et al., 2013, 2017, 2019).

We obtained and evaluated Sentinel‐3A OLCI Level 2 image products with 300 × 300 m water pixels in the IRL study area from the EUMETSAT Copernicus data repository. A total of 12 images between 7/31/17 and 1/1/19 with minimal cloud cover at the Sykes Creek Kilroy location (Figure 1b), located within the Banana River region of the IRL, were selected for analysis (Table 1). The June 28, 2018, image acquired 1 to 2 days before the 2‐day field sampling work on 6/29/18 to 6/30/18 was used for comparison with field observations due to its near coincident timing and minimal cloud coverage. Using Harris Geospatial ENVI/IDL software, the center‐weighted derivative for the 11‐visible bands in the Sentinel‐3A OLCI spectra were calculated for the data from each image following the numerical method presented in Press et al. (1992).

To isolate the signal associated with the brown tide bloom, we conducted separate VPCA on the ASD Fieldspec® HH2 field data and the Sentinel‐3A OLCI derivative spectra datasets obtained from each of the 12 images. VPCA reduces the dimensionality of multivariate datasets by decomposing the integrated spectral signatures into orthogonal axes that each account for a portion of the total signal variability (Ortiz et al., 2019). In this way, it removes redundant information from the image. Following the varimax rotation, the results yield component loadings, which represent independent spectral signatures of constituents in the water column, and component scores, which represent the spatial distribution of these spectral signatures. For each day, the components are sorted in variance rank order, but the variance explained by each component can vary with time. As a result, component rank is not diagnostic of composition. For this reason, we match the numerically ranked components extracted from our calibration image (6/28/18) with the components for the remaining days in the data set into component groups designated as Patterns A, B, C, and D, which correspond to the repeating, independent spectral signals extracted from the images. For satellite images, the component scores were displayed as distribution maps, which represent the fractional variance associated with each component at each pixel, while the component scores of the field spectra represent single location values at the field sampling site. These orthogonal VPCA component loadings and scores for the multispectral satellite imagery were calculated using code written for ENVI/IDL (Avouris & Ortiz, 2019; Ortiz et al., 2017, 2019). The hyperspectral spectroradiometer data was processed in SPSS using the same methodology.

### 3.6 Validation and Spectral Identification

The VPCA component loadings obtained for all datasets were identified by forward stepwise principal component regression against a spectral constituent library (Avouris & Ortiz, 2019; Ortiz et al., 2019), with the inclusion of the additional spectra measured from the filtered *A*. *lagunensis* culture. We also correlate the extracted VPCA spectra directly with the reference spectrum for *A*. *lagunensis* measured from the culture. The library of known reflectance derivative spectra for water quality constituents includes 84 signatures: 10 for algal groups or taxa, 27 extracted algal and cyanobacterial pigments and accessory pigments, six chlorophyll a and b degradation products, and 41 minerals compiled from the literature (Ortiz et al., 2013, 2019 and references therein), using mineral spectra from the United States Geological Survey (USGS) Spectral Library (Kokaly et al., 2017) or measured in the lab (Ortiz et al., 2013, 2019).

Use of principal component regression addresses the main weakness of stepwise multiple linear regression and provides robust results because the signals extracted from the images are independent, minimizing the potential for multicollinearity in the results. In addition to standard regression statistics (R, R^2^, *F* value, *p* value), the level of multicollinearity on a term by term basis was assessed using the variance inflation factor (VIF). The individual VPCA loadings were fit to as many matching spectral constituents in the library as needed before reaching a stopping criterion of VIF **≤** 2 for all terms in the equation, rather than the customary value of 4–5. This approach minimizes the risk of over fitting (Ortiz et al., 2019). The VIF values are reported as the range of calculated values when different, or as a single value if identical VIFs were found for all terms. Even with a VIF threshold as low as 2, statistically significant fits that explain the majority of variance in the VPCA components (*R* ^2^ **≥** 0.87) were obtained with mixtures of only two to three constituents due to the orthogonal nature of the principal components. Once the spectral signatures of all component loadings were identified using the constituent library, the component loadings or scores for the two instrument datasets were correlated against each other. Correlations between the two datasets was useful to demonstrate consistency of the KSU VPCA spectral decomposition method across the hyper‐ and multispectral instruments. To further validate spectral identifications with *A*. *lagunensis* and other potential constituents, we correlated the spatially related, component scores from the field instrument and extracted component score pixel values from the VPCA distribution map of the Sentinel‐3A image to the phylum‐level cell density and biovolume results obtained for each sample site and the YSI EXO2 sonde parameters.

This approach addresses the mixed pixel problem for optically complex water by separating out image variance due to extraneous factors such as bottom reflectance, suspended sediment, CDOM, or other types of algae and cyanobacteria or random noise from the signature of interest, in this case the spectral response of *A*. *lagunensis* or related species. We present the spectral shapes and identification for four components extracted from our validation image but focus on the signature of the brown tide‐related component for the remaining images because the focus of this applied geohealth‐related paper is assessment of the HAB by remote sensing. The other extracted VPCA components could, however, be used to provide information regarding the distribution of suspended sediment, the distribution of pigment degradation products, or to assess changes in algal community structure with space and time. Topics that can be discussed in future publications.

### 3.7 Comparison with Prior Studies: Chl a Estimation

Prior remote sensing studies of the IRL reconstructed bloom intensity and extent using Chl a as a proxy for biomass. Kamerosky et al. (2015) applied the red edge band ratio algorithms of Moses et al. (2009) and Mishra & Mishra (2012) to estimate Chl a in the IRL during the 2011 super bloom. The algorithm of Mishra & Mishra (2012) is referred to as the Normalized Chlorophyll Difference Index (NDCI). The algorithms are two‐band Red‐NIR algorithms that make use of a band centered on the red edge to monitor Chl a, which is normalized against an NIR band assumed to have minimal interference from Chl a. These algorithms were originally devised for Chl a estimation in the Sea of Azov (Moses et al., 2009) and Chesapeake, Delaware, and Mobile Bays and the Mississippi Delta (Mishra and Mishra (2012), all of which are optically complex estuarine or marginal marine environments prone to harmful algal blooms. Kamerosky et al. (2015) regionally tuned the algorithms for use in the IRL by calibration with a data set of 50 samples and then validated them using an additional 40 independent samples that were withheld from the calibration analysis. This study compares against their calibration results.

For comparison with Kamerosky et al. (2015), we built three simple principal component regressions from the extracted VPCA components, calibrated with the nine Chl a measurements from the YSI EXO sonde collected at the cloud‐free locations of our field samples. The dependent variable for the first model was the weighted sum of the Sentinel‐3A VPCA scores, where the weighting factor was the fractional variance explained by each of the three components that exhibited a red‐edge response (VPCAs 2, 3, and 4 or Patterns B, C, and D). For this first model, VPCA 1 (Pattern A), which did not exhibit a red‐edge response and which was identified as primarily related to the mineral illite was excluded. We compared linear and quadratic fits to the Chl a data for the first and second model for direct comparison with the results from Kamerosky et al. (2015). For comparison purposes and to show the importance of the spectral decomposition, we also constructed a third model, which was based on the variance‐weighted sum of VPCAs 1 to 4 (Patterns A, B, C, and D), which was regressed against the field Chl a data.

## 4 Results

### 4.1 ORCA Kilroy Chl a and BGA‐PE Time Series

**Figure 2:**
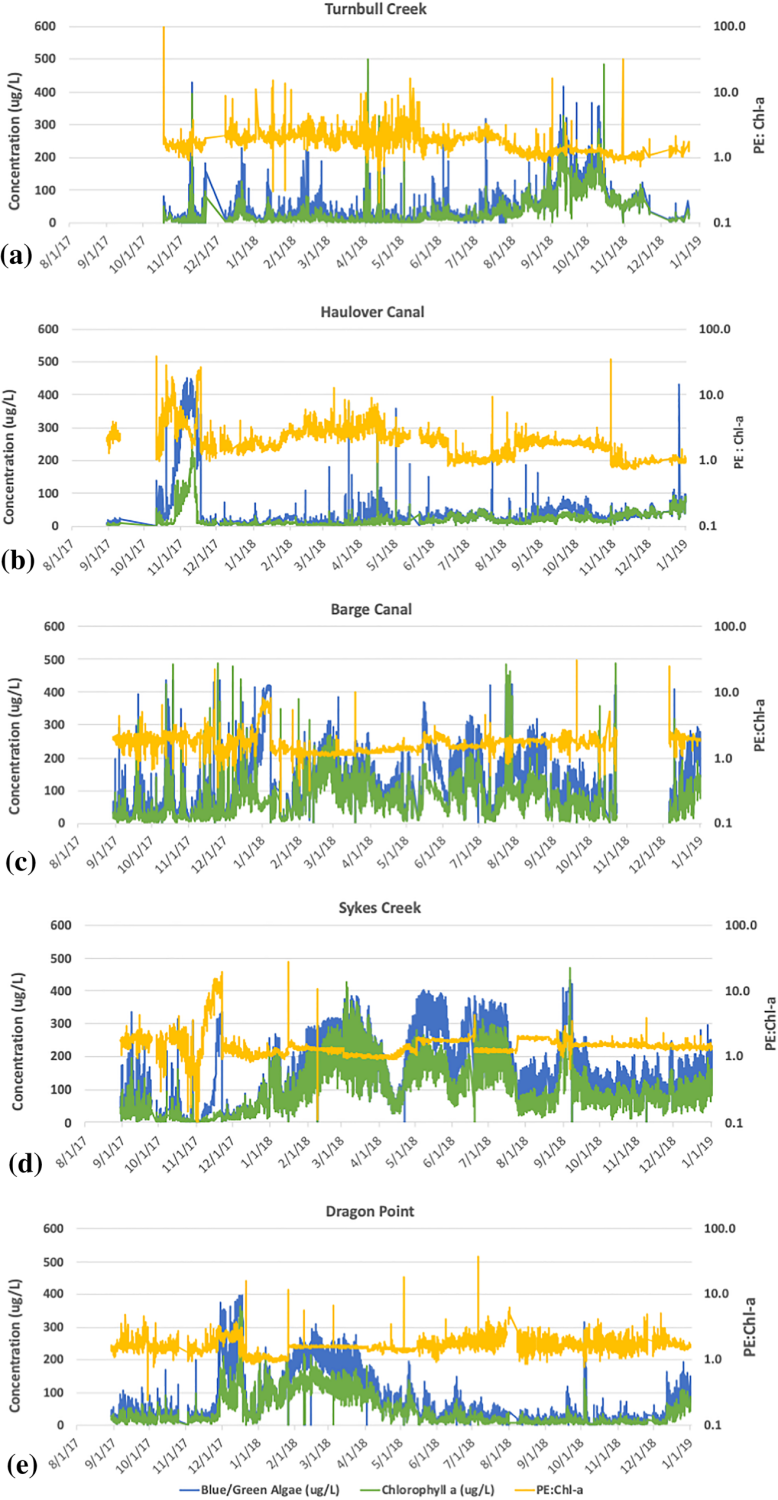
Time series of BGE‐PE (blue) and Chl a (green) and PE:Chl a (yellow) from 8/1/2017 to 1/1/2019 (http://api.kilroydata.org/public/) for (a) Turnbull Creek, (b) Haulover Canal, (c) Barge Canal, (d) Sykes Creek, and (e) Dragon Point.

The Chl a and BGE‐PE concentrations obtained from the Kilroy environmental monitoring systems provided time series of measurements that indicate the variability and the bloom extent during the span of ~1.5 years (Figure 2). The Chl a and BGE‐PE values were generally low at the two northern stations (Figure 2a,b: Turnbull Creek and Haulover Canal), with occasional increases in Chl a, BGE‐PE, and the PE:Chl a ratio to values above 5. The Chl a and BGE‐PE were generally higher at the Barge Canal and Sykes Creek stations (Figure 2c, d) but with PE:Chl a generally below 5. At the Sykes Creek Kilroy site within the Banana River (Figure 2d), where high Ochrophyta counts are detected, concentrations range between 0–389 μg/mL for BGE‐PE and 0–427 μg/mL for Chl a (Figure 2d), with PE:Chl a ratios that are generally between 1 and 5, but which can reach values are high as 19. The Chl a and BGE‐PE values were generally low at the Dragon Point station (Figure 2e), except for one period from January to April 2018 when values increased in concert with the signal from the Sykes Creek station.

### 4.2 Visible Derivative Spectroscopy and VPCA Spectral Identifications

**Figure 3:**
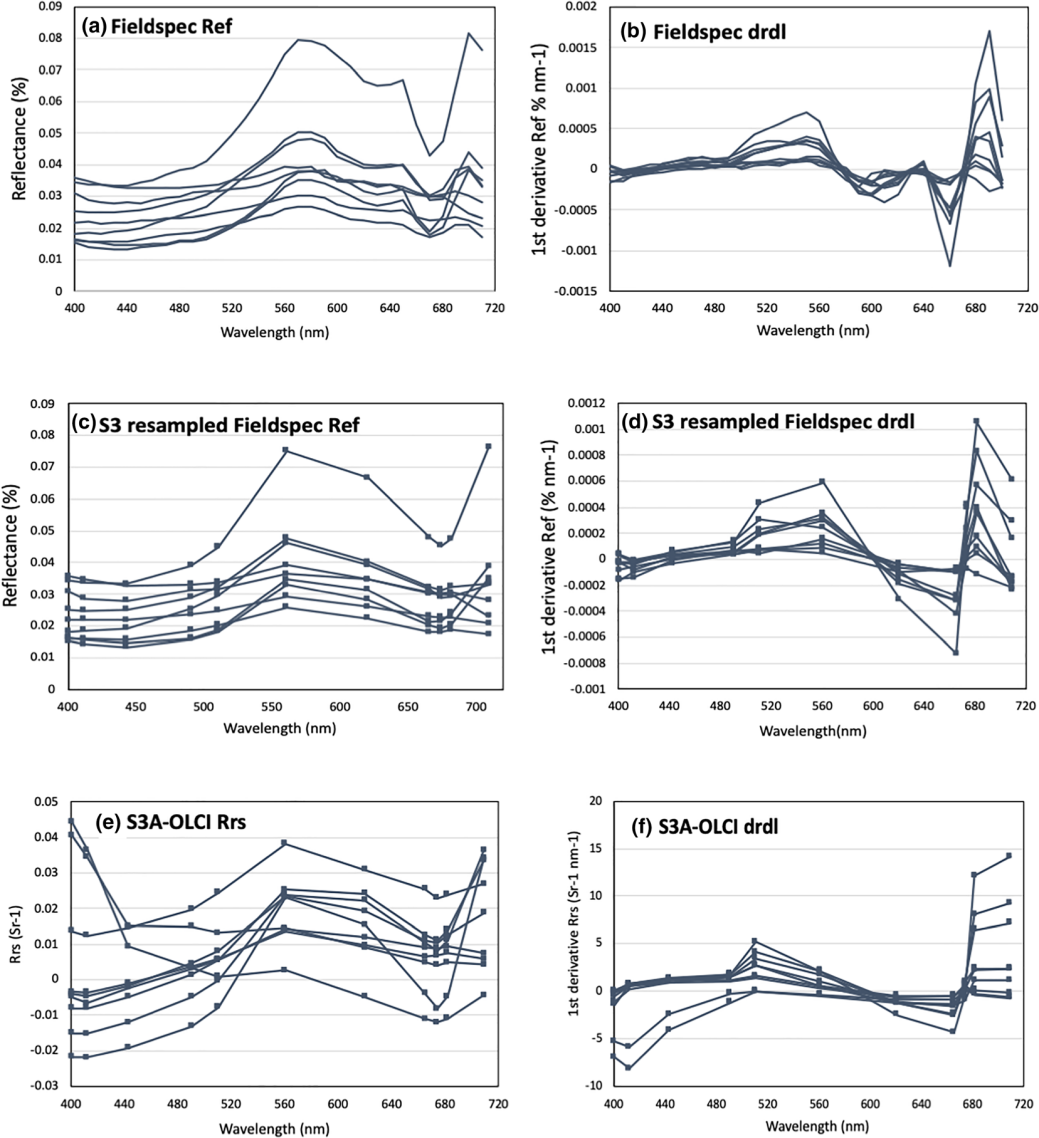
Analytical Spectral Devices™ (ASD) FieldSpec® HH2 reflectance (a) and derivative (b) spectra from field sites on the Indian River Lagoon collected between 6/29/2018 and 6/30/2018. The same ASD FieldSpec® HH2 data resampled to Sentinel‐3A OLCI spectral resolution for reflectance (c) and derivative (d) spectra. These resampled spectra are compared with Level 2 Sentinel‐3A OLCI spectral retrieval from 6/28/2018 as reflectance (e) and derivative (f) spectra.

The reflectance spectra obtained with the ASD FieldSpec® HH2 and from the Sentinel‐3A imagery document that the IRL is an optically complex environment (Figure 3). The ASD FieldSpec® reflectance exhibit a broad reflectance trough from 400 to 520 nm, a broad reflectance maximum from 560 to 600 nm, with a shoulder from 620 to 660 nm, and a sharp reflectance trough centered on 680 nm before reaching an overall maximum at 700 nm. These features can be related to absorption by suspended sediment or CDOM on the blue end of the spectrum, accessory pigments and iron oxides in the yellow and green, and cyanobacterial pigments such as phycocyanin or phycoerythrin and of course Chl a in the orange and red portions of the spectrum. To remove the influence of scattering we transform the reflectance spectra (Figure 3a, c, e) to derivative spectra (3b, 3d, 3f). To allow for more direct comparison to Sentinel‐3A OLCI spectra, we resampled the hyperspectral ASD FieldSpec® HH2 spectra to OLCI band resolution (Figure 3c, d). The combination of derivative transformation and resampling produces ASD FieldSpec® HH2 derivative spectra (Figure 3d) that bear more resemblance to the Sentinel‐3A OLCI derivative spectra (Figure 3f) than the original reflectance spectra (Figure 3c, 3e), demonstrating one of the strengths of the pre‐processing steps we incorporate into the VPCA method.

The derivative spectra presented in Figure 3d, and Figure 3f and the other pixels in the Sentinel‐3A OLCI images still represent mixtures of multiple constituents. To partition that variance, we used the VPCA method to yield independent components. An advantage of this approach is that it provides two ways in which the results can be interpreted. We match the extracted component loadings with spectra from a known library to spectrally *calibrate* the images by determining the identity of each component then *validate* the images by comparison of independent field observations against the spatial component scores extracted from the June 28, 2018, image. We present the spectral calibration in the remainder of this section and the spatial field validation in Section 4.3.

**Figure 4:**
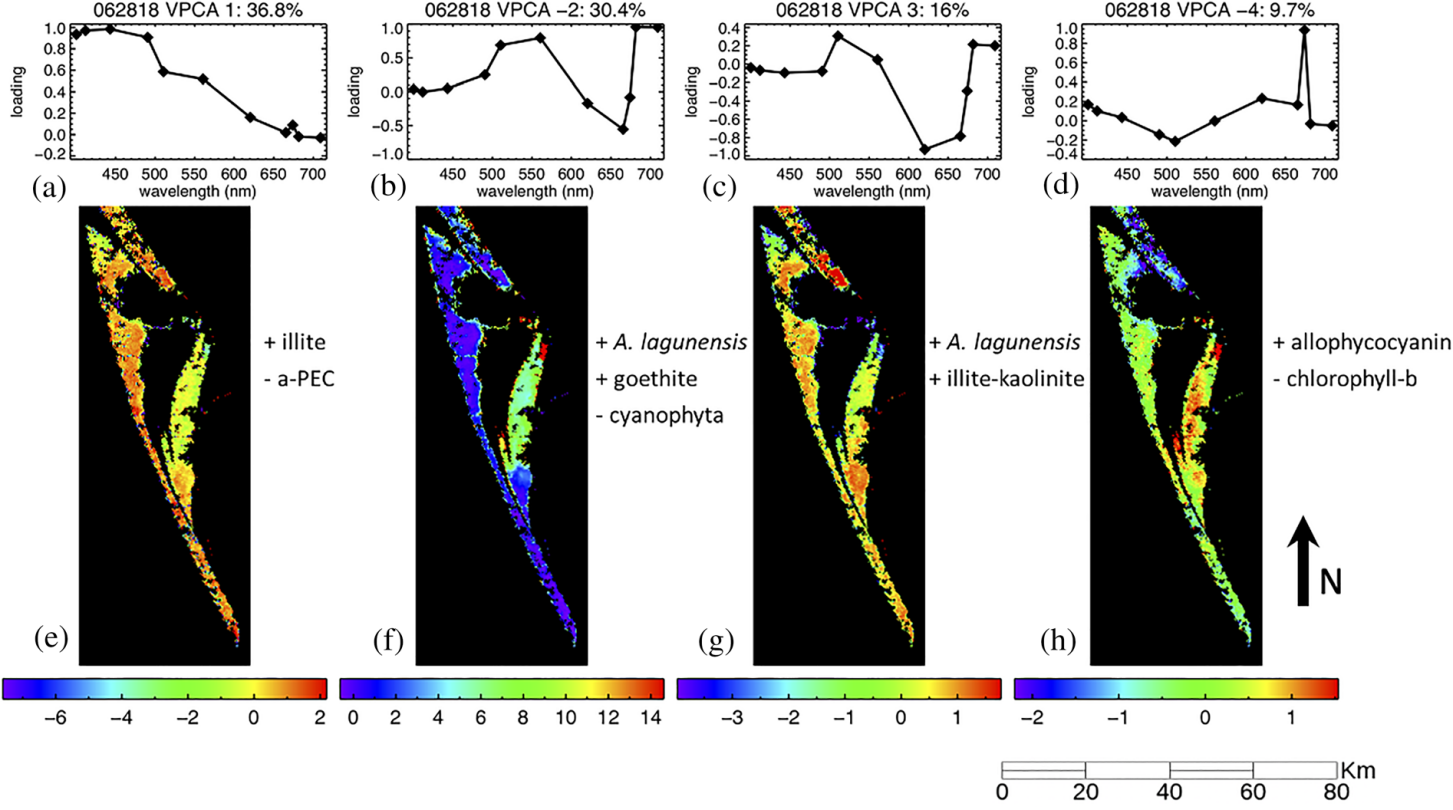
Indian River Lagoon spectral (a–d) and spatial distribution maps (e–h) of component loadings from June 28, 2018, swath from Sentinel‐3A OLCI. In all images, warm colors indicate higher association of the pixels with the spectral constituent mixture represented by this pattern while cool colors indicate a lower association. Components 1 to 4 correspond to Patterns A to D when matched and averaged across the 12 images.

The results from the VPCA spectral decomposition of Sentinel‐3A imagery and HH2 field measured spectra both yield four unique VPCs (Table S2) that are similar but not identical due to differences in spatial sampling and atmospheric errors. For each VPC, the loading patterns were regressed and matched with known spectral signatures from a spectral library. The results from the June 28, 2018, image are described as representative. In total, the components from the Sentinel‐3A OLCI decomposition account for 92.9% of the total signal variability (Figure 4). Sentinel‐3A OLCI VPC loading 1 accounts for 36.8% of the total image variability (Figure 4) and has a positive correlation with illite and negative correlation with alpha‐phycoerythrocyanin (α‐PEC), a cyanobacterial pigment (*R* = 0.98, *R* ^2^ = 0.96, VIF = 1.0, *p* = 1.4 × 10^−6^, *df* = 8). Components 2 and 3 have a positive correlation with *A*. *lagunensis* (Figure 4b, 4c) but account for its association with different mixtures of in water optical constituents. Component 2 accounts for 30.4% of image variability with a positive correlation between *A*. *lagunensis*, and goethite, but a negative correlation with a cyanophyta pigment signature (*R* = 0.99, *R* ^2^ = 0.98, VIF = 1.2 to 1.8, *p* = 7.6 × 10^−7^, *df* = 7). Component 3 correlates with *A*. *lagunensis* and illite + kaolinite, but accounts for a smaller, 16% of the image variability (*R* = 0.85, *R* ^2^ = 0.72, VIF = 1.0, *p* = 0.053, *df* = 8). Component 4 (Figure 4d) has a positive correlation with allophycocyanin, a cyanobacterial pigment, and a negative correlation to Chlorophyll b, accounting for 9.7% of the signal variability (*R* = 0.82, *R* ^2^ = 0.67, VIF = 1.5, *p* = 0.0118, *df* = 8). The individual components, which represent the partitioned variance from the combined in‐water optical constituents, can be linked to their spatial distribution within the IRL as component scores (Figure 4e–h). Based on the spatial distribution map, Components 2 and 4 were highly concentrated in the Banana River region of the IRL.

**Figure 5:**
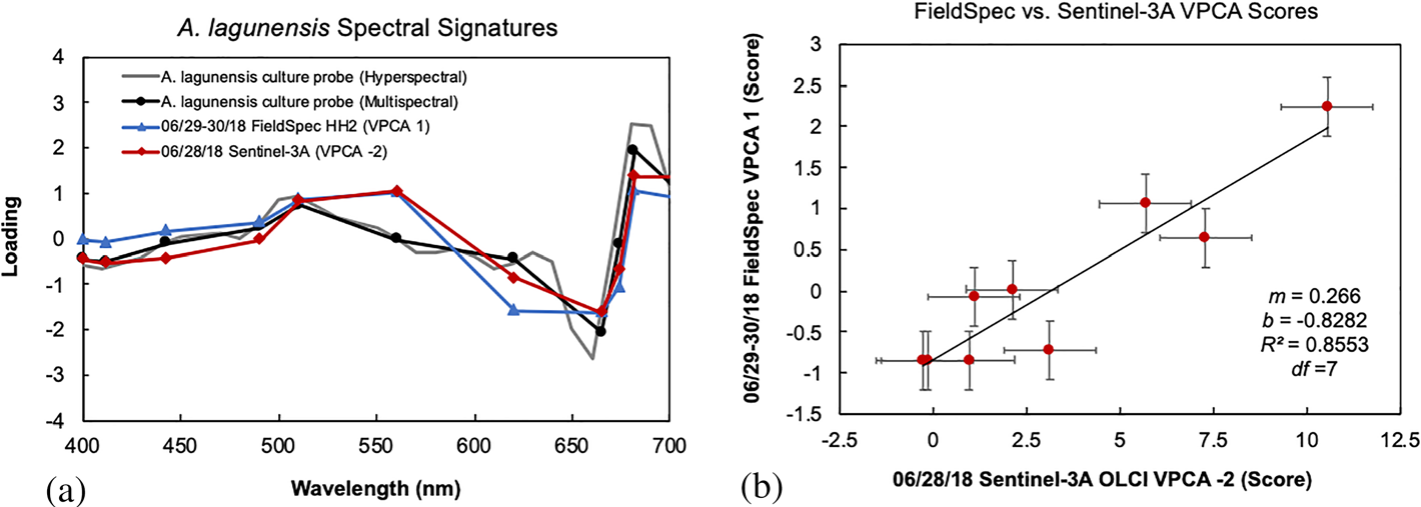
Validation graphs showing (a) spectral signature of the *Aureoumbra lagunensis* culture compared with varimax‐rotated principal component analysis (VPCA) component loadings for both satellite and field measured datasets at the 11‐band resolution of Sentinel‐3A OLCI. (b) Comparison between the component scores of field spectra and Sentinel‐3A that represent constituents of *A*. *lagunensis*.

The spectral shapes of the OLCI VPC 2 loading exhibits similar absorption peaks to lab measured *A*. *lagunensis* cultures and the VPC 1 loading of the ASD HH2 spectra (Figure 5a). Comparing the results of the two independent VPCA spectral decompositions with the signature from the lab measured *A*. *lagunensis* culture, we find a significant correlation between the spectral shapes of the component loadings collected with the hyperspectral ASD HH2 VPCA 1 (*R* = 0.77, *R* ^2^ = 0.59, *p* = 0.0059, *df* = 9) and the OLCI VPCA 2 component extracted from the multispectral Sentinel‐3A OLCI imagery (*R* = 0.87, *R* ^2^ = 0.75, *p* = 0.00049, *df* = 9). The spectral signals from the ASD HH2 VPCA 1 and the OLCI VPCA 2 are also highly correlated to each other (*R* = 0.91, *R* ^2^ = 0.83, *p* = 0.00012, *df* = 9).

Although measured with different instruments and at only a subset of the locations within the IRL sampled by Sentinel‐3A OLCI, the four component loadings produced from field data collected with an ASD FieldSpec® HH2 at near coinciding locations exhibit similar constituent identifications and spatial patterns to those found with the Sentinel‐3A OLCI analysis (Table S2). The HH2 VPC 1 loading accounts for 48.4% of total signal variability and has a positive correlation with *A*. *lagunensis* and kaolinite (*R* = 0.89, *R* ^2^ = 0.79, VIF = 1.0, *p* = 0.0027, *df* = 8). HH2 VPC loading 2 accounts for 31% of variability across all sites and has a positive correlation with illite and allophycocyanin (*R* = 0.93, *R* ^2^ = 0.86, VIF = 1.2, *p* = 0.0004, *df* = 8). HH2 VPC 3 loading accounts for 10.5% of variability and has a positive correlation with α‐PEC and illite + kaolinite (*R* = 0.83, *R* ^2^ = 0.68, VIF = 1.0, *p* = 0.0069, *df* = 8). HH2 VPC loading 4 accounts for 5% of variability and has a positive correlation with α‐PEC and Chlorophyll‐a + carotenoids (*R* = 0.91, *R* ^2^ = 0.83, VIF = 1.0, *p* = 0.0008, *df* = 8).

### 4.3 Field Spatial Validation

**Figure 6:**
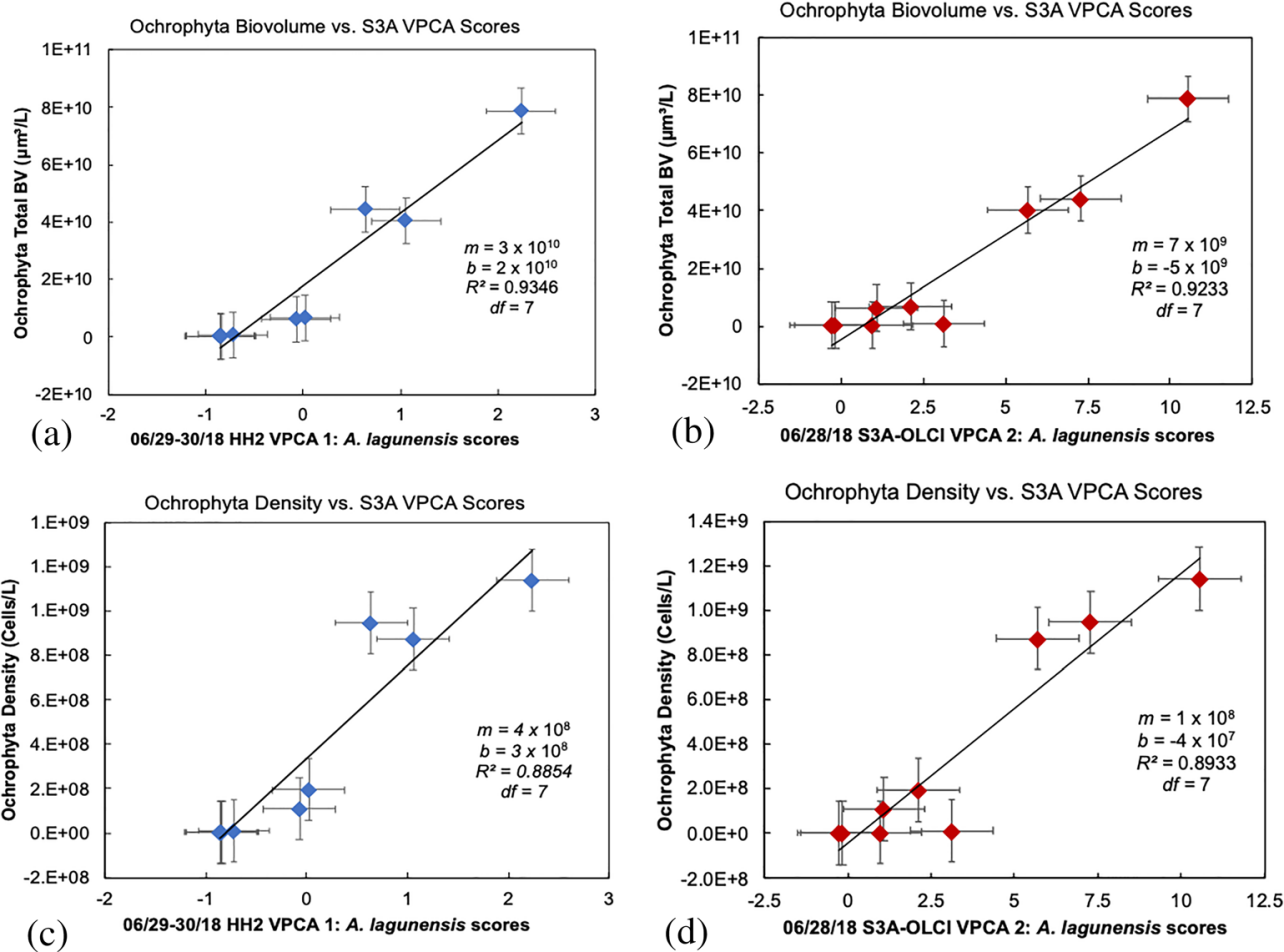
Validation graphs showing the comparison between (a) HH2 varimax‐rotated principal component analysis (VPCA) component scores and (b) Sentinel‐3A OLCI VPCA component scores with Ochrophyta biovolume (μm^3^/L) and (c) HH2 VPCA component scores and (d) Sentinel‐3A OLCI VPCA component scores with Ochrophyta cell density (cells/L).

When the two spectroradiometers were sampled at their common locations (Figure 1b), despite their different spatial scales (300 m vs. ~50 cm), the spatial pattern of the retrievals from the OLCI sensor and the HH2 were highly statistically correlated (Figure 5b), with *R* = 0.92 (*R* ^2^ = 0.86, *df* = 7, *p* = 3.6 × 10^−4^). The sample locations that had the highest cell count density and biovolume for Ochrophyta were found in the Banana River. When comparing the biovolume estimates from cell counts, with the VPCA component scores, we find a positive correlation for Ochrophyta with *A*. *lagunensis* and the HH2 field spectra VPC 1 (Figure 6a) with *R* = 0.97 (*R* ^2^ = 0.93, *df* = 7, *p* = 2 × 10^−5^) and with Sentinel‐3A OLCI VPCA 2 (Figure 6b), with *R* = 0.96 (*R* ^2^ = 0.92, *df* = 7, *p* = 4 × 10^−5^). The response between the biovolume estimates and component scores was linear from biovolumes of 0 to 8 × 10^10^ μm^3^/L. The HH2 and Sentinel‐3A OLCI VPCA results were also highly correlated with the cell counts when the data were compared as cell density in cells per liter (Figure 6c, d).

Nine stations were cloud free during field operations for comparison of the OLCI VPCA components and the mobile YSI EXO2 sonde profile measurements (see full correlation results in Table 2). Averaging the YSI EXO2 sonde profiles over the first optical depth (Table S1) documents that OLCI VPC 1 was highly positively correlated with the PE:Chl a ratio. Component OLCI VPCA −2 was inversely corelated with specific conductivity, salinity, and Secchi depth, but highly positively correlated with turbidity, and positively correlated with pH. Two of the OLCI components were positively correlated with Chl a and BGA‐PE. OLCI VPC −2 exhibited a stronger correlation with Chl a (*R* = 0.94, *R* ^2^ = 0.89, *p* = 0.0014, *df* = 7) and a weaker correlation with BGE‐PE (*R* = 0.75, *R* ^2^ = 0.56, *p* = 0.020, *df* = 7). The same pattern was observed for OCLI VPC 4 with respect to Chl a (*R* = 0.75, *R* ^2^ = 0.56, *p* = 0.021, *df* = 7) and BGE‐PE (*R* = 0.75, *R* ^2^ = 0.56, *p* = 0.019, *df* = 7).

**Table 2:** Field Calibration Intercomparisons

Comparison	*R*	*R* ^2^	*p*	*df*
Ochrophyta cell biovol. vs. ASD Fieldspec HH2 VPCA 1	0.97	0.93	2 × 10^−5^	7
Ochrophyta cell biovol. vs. S3AOLCI VPCA −2	0.96	0.92	4 × 10^−5^	7
ASD Fieldspec HH2 VPCA 1 vs. S3AOLCI VPCA −2	0.92	0.86	3.6 × 10^−4^	7
Secchi depth vs. S3AOLCI VPCA −2	−0.74	0.55	0.022	7
YSI PE:Chl a vs. S3AOLCI VPCA 1	0.84	0.70	4.8 × 10^−3^	7
YSI Specific conductivity (μS/cm) vs. S3AOLCI VPCA −2	−0.67	0.45	0.047	7
YSI Salinity vs. S3AOLCI VPCA −2	−0.67	0.45	0.049	7
YSI Turbidity vs. S3AOLCI VPCA −2	0.88	0.77	1.9 × 10^−3^	7
YSI pH vs. S3AOLCI VPCA −2	0.75	0.56	0.021	7
YSI Chl a vs. S3AOLCI VPCA 1	0.08	0.01	0.831	7
YSI Chl a vs. S3AOLCI VPCA −2	0.94	0.89	1.4 × 10^−3^	7
YSI Chl a vs. S3AOLCI VPCA 3	−0.32	0.10	0.40	7
YSI Chl a vs. S3AOLCI VPCA 4	0.75	0.56	0.021	7
YSI Chl a vs. Weighted sum S3AOLCI VPCA 2 to 4	0.96	0.92	4.0 × 10^−5^	7
YSI Chl a vs. Weighted sum S3AOLCI VPCA 1 to 4	0.83	0.69	5.3 × 10^−3^	7

Our component loading identification results show that it is possible to isolate the *A*. *lagunensis* signal, with the inclusion of some minor suspended sediment constituents, from the integrated spectral signature of the optically complex water and thus identify the region of the IRL most affected by brown tide. The second component effectively isolated the spatial distribution of *A*. *lagunensis* and cyanobacteria in the various subbasins of the IRL. Where this mode exhibits strong positive scores, *A*. *lagunensis* is found in greater abundance and where it exhibits strong negative loadings, *A*. *lagunensis* is found in lower abundance, with cyanobacteria in higher abundance. This linear relationship holds because the component scores represent true partial correlations, with positive scores indicating standard deviations greater than the mean and negative scores indicating standard deviations lower than the mean. Because the data set is centered at the beginning of the analysis, the mean value is equal to zero in this case.

The identification of the brown tide is possible even when the *A*. *lagunensis* signal represents only ~30% of the total image variance, despite the fact that the bloom has reached considerable size in terms of Chl a. The VPCA method also provides reliable results across several orders of magnitude in cell density and biovolume with intercepts that are not statistically different from zero. These results thus enable brown tide monitoring to identify blooms at an early stage, providing more time for stakeholder response to this public health problem. The presence of matching VPCA components obtained from both the ASD FieldSpec® HH2 and Sentinel‐3A OLCI decomposition further validates these results. The relative ranking of the component in the OLCI and HH2 data set change because the components are extracted on data sets of different sizes with measurements collected at slightly different times, at differing spectral resolution, and with different amounts of atmospheric error. Even when the HH2 component loadings were downgraded to the Sentinel‐3A OLCI's 11‐band spectral resolution, we still obtain statistically significant correlations between similarly identified components, specifically, the HH2 VPC 1 loading compared with OLCI VPC 2 for *A*. *lagunensis* (Figure 5a,b). In all, the three spectral and three spatial patterns from three independent reflectance derivative data sets (*A*. *lagunensis* culture spectra, ASD HH2 field spectra, and Sentinel‐3A OLCI sensor data) are all statistically correlated to each other and to the independent cell count density and biovolume results, documenting the high quality of the satellite retrievals (Table 2). The *p* values for these seven relationships (four spectral and three spatial) are all highly significant, ranging from *p* = 7.6 × 10^−7^ to *p* = 2.7 × 10^−3^. The joint probability for all seven of these relationships to be observed in concert by random chance alone is vanishingly small.

### 4.4 VPCA Spatial–Temporal Variability of the Banana River Brown Tide Bloom

**Figure 7:**
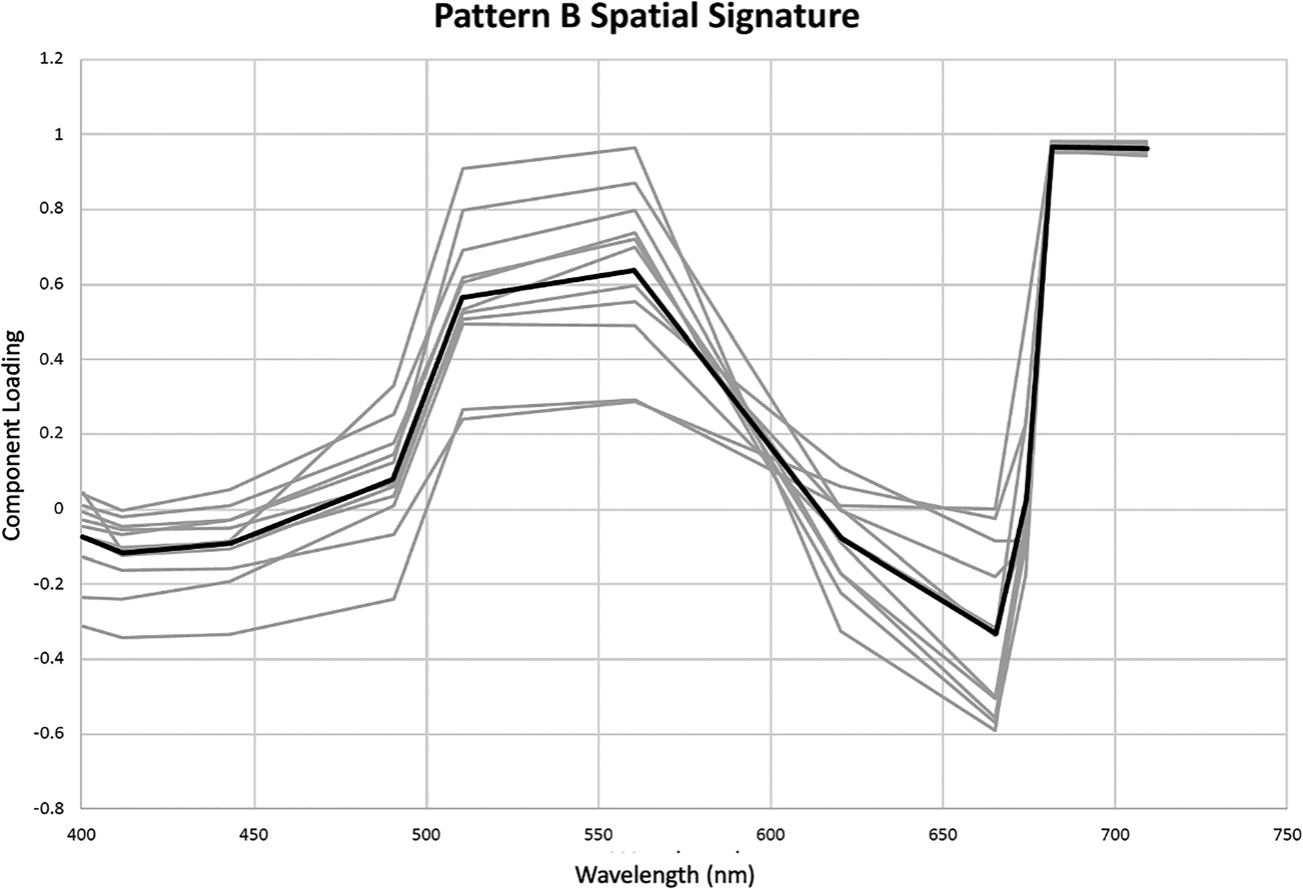
Average spectral shape (thick black curve) and daily variability (thin gray curves) for varimax‐rotated principal component analysis Pattern B loadings, which relates to Ochrophyta, *Aureoumbra lagunensis*, and the brown tide in the Indian River Lagoon. The daily spectra correspond to the components extracted from the 12 images between August 1, 2017, to November 21, 2018.

**Figure 8:**
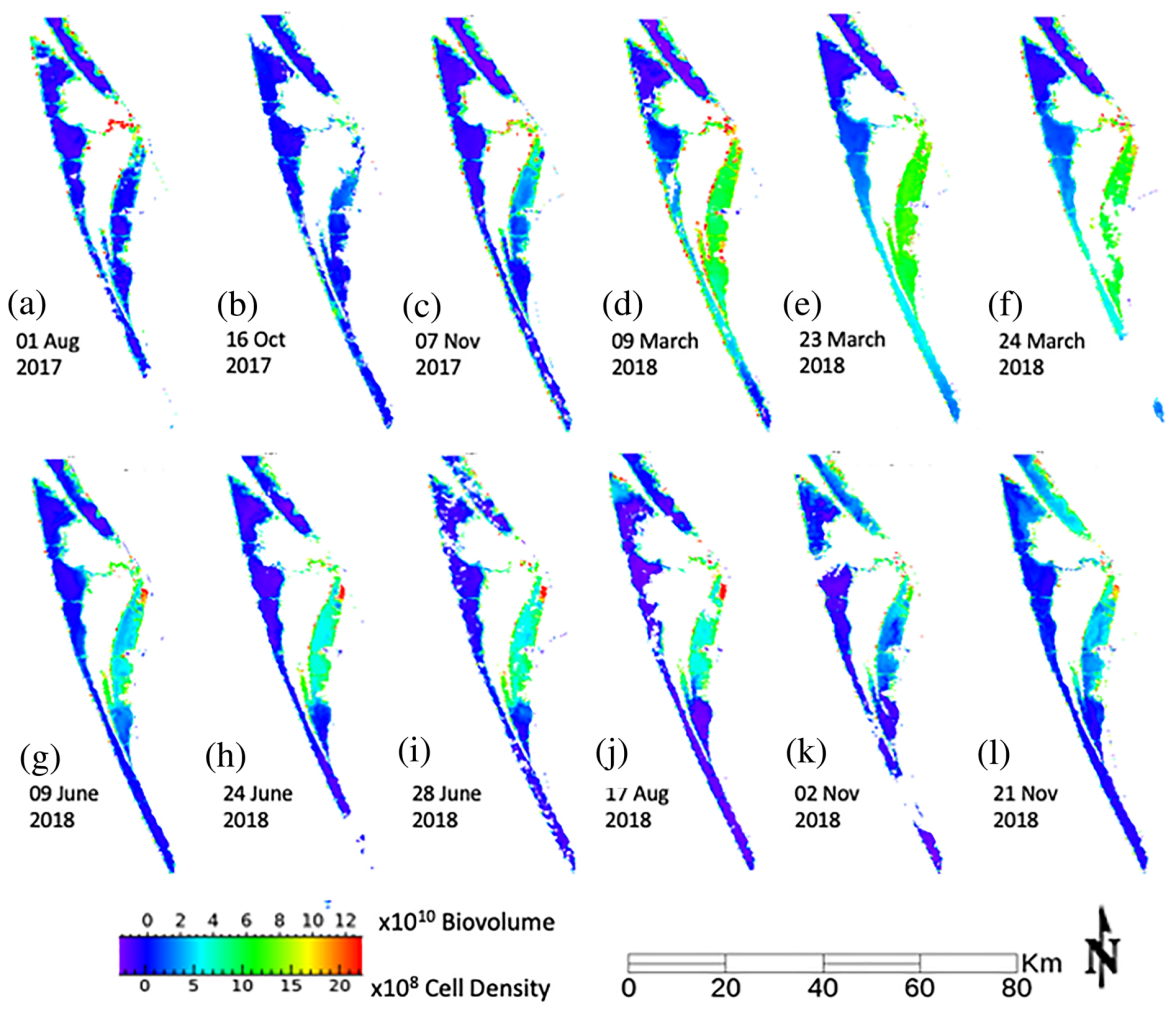
Time series from August 1, 2017, to November 21, 2018, of 12 spatial (a‐l) distribution maps for average VPC Pattern B scores, which represents spectral constituents that correlate positively to the brown tide signature. In all images, warm colors indicate higher biovolume (μm^3^/L) or cell density (cells/L) while cool colors indicate a lower values.

The same spectral shape, associated with the *A*. *lagunensis* bloom, identified as VPCA Pattern B can be extracted from each of the Sentinel‐3A OLCI images studied (Figure 7). These 12 Sentinel‐3A OLCI images (Figure 8) document variations in the spatial extent and intensity of the brown tide that are consistent with the variations in the magnitude of the bloom measured by the Chl a and BGA‐PE time series recorded by the five Kilroy sensors at Turnbull Creek, Haulover Canal, Barge Canal, Sykes Creek, and Dragon Point (compare Figure 2a‐e with Figure 8a‐l). To make the results presented in Figure 8 as useful as possible to water quality managers, the maps were scaled in terms of biovolume and cell density by regressing the component scores extracted from each image using the average spectral response for Sentinel‐3A OLCI VPCA Pattern B against the cell count data. The relationships employed have a slope and intercept of 6.8 × 10^9^ and −3.8 × 10^9^ for biovolume in μm^3^/L (*R* = 0.98, *R* ^2^ = 0.96, *p* = 5.6 × 10^−6^, *df* = 7) and a slope and intercept of 1.1 × 10^8^ and −3.0 × 10^7^ for cell density in cells/L (*R* = 0.97, *R* ^2^ = 0.94, *p* = 2.0 × 10^−5^, *df* = 7). Notice that these coefficients are consistent within error with the Sentinel 3A OLCI regressions calculated based on the individual daily results for 6/28/18 (Figure 6b, d), demonstrating the stability of the coefficients obtained by principal component regression.

The coarse temporal sampling of the Sentinel‐3A OLCI images aliases the daily to weekly variability observed in the Sykes Creek time series but is consistent with the time series values that were collected coincident with the images. The bloom was at a minimum extent on 8/1/17, with most values in the three sub‐basins below ~2 × 10^10^ μm^3^/L or ~4 × 10^8^ cells/L, which are below the limits for a “minor bloom” as defined by the USEPA (USEPA, 2016), but increased in extent in the discrete images to a maximum between 3/9/18 through 3/24/18, with values in much of the Banana River at or exceeding ~6 × 10^10^ μm^3^/L or ~10 × 10^8^ cells/L, which meet the USEPA criteria for a “severe bloom” in terms of cell density, then decreased in extent from 6/9/19 to 11/21/18 to values near ~2.5 × 10^10^ μm^3^/L or ~5 × 10^8^ cells/L, which are consistent with the USEPA guidelines for a minor bloom, which persisted throughout that time. As indicated by Figure 2, the brown tide was generally not as extensive at Turnbull Creek or Haulover Canal in the Northern Indian River as was observed at Sykes Creek in the Banana River. At Turnbull Creek and Haulover Canal, the spatial pattern for OLCI VPCA 2 also increased towards the end of the time series (Figure 8a‐l). The signal at Haulover Canal (Figure 2b) increased from very low Chl a, BGA‐PE values at the start of the ~1.5‐year period to values that averaged ~200 μg/L with spikes over 400 μg/L. Values at the Haulover Canal Kilroy sensor were generally much lower, averaging between 0 and 40 μg/L for most of the record with occasional spikes to 80 μg/L or greater (Figure 2b). A notable exception to this pattern at Haulover Canal was a brief bloom between October and November 2017, which reached BGA‐PE as high as 440 μg/L, but Chl a of only 280 μg/L (Figure 2b). This bloom was thus associated with PE:Chl a ratios >10, suggesting that it was not a brown tide event, but rather a cyanobacterial bloom. The signal at Barge Canal (Figure 2c) matched that at Sykes Creek (Figure 2d) during times when the PE:Chl a ratio varied between 1 and 5 but also exhibited brief blooms with PE:Chl a >10 that were not registered at Sykes Creek. Additional documentation for the conclusion that PE:Chl a ratios >10 are indicative of cyanobacterial blooms is provided by the fact that while the OLCI VPCA Pattern B did not register the high PE:Chl a event at Haulover Canal (e.g., Figure 8) or the high PE:Chl a events at Barge Canal, they were picked up as increases in OLCI VPCA Pattern D, which has been identified as positively correlated with the cyanobacterial pigment allophycocyanin and inversely correlated with Chlorophyll‐b in the images analyzed from that time period (Judice, 2019). The sensors at Dragon Point recorded the two‐pulsed extension of the bloom from the Banana River into the central IRL from 12/17 to 4/18. This event was observed in the two Sentinel‐3A OLCI images collected on 3/9/18 (Figure 8d) and 3/23/18 (Figure 8e) but partially obscured by clouds in the 3/24/18 (Figure 8g) image.

## 5 Discussion

### 5.1 Remote Sensing of HABs

Remote sensing studies of HABs are often based on estimation of Chl a, which provides a measure of the overall bloom intensity. However, many HABs include complex community structures (Binding et al., 2019; Hall et al., 2013, 2018; Paerl et al., 2008) and while toxic and nontoxic strains of HAB forming cyanobacteria all contain Chl a, there is evidence that they exhibit different accessory pigments or pigment ratios in the lab (Akins et al., 2020; Islam & Beardal, 2017). Accordingly, Chl a is not diagnostic of HAB conditions and more specific tools are needed to improve HAB detection, an important public health need.

**Figure 9:**
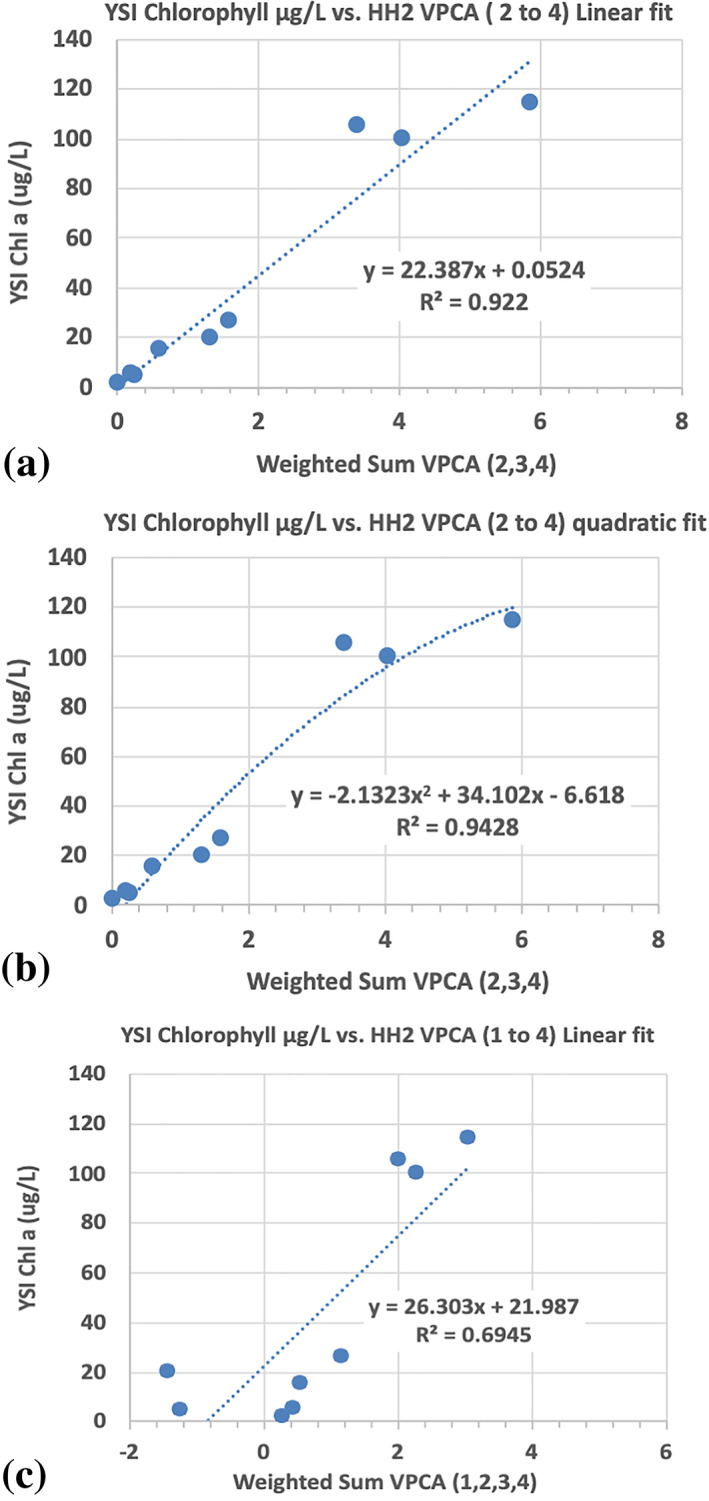
Regression fits for Chl a based on (a) model 1: linear fit to S3A OLCI varimax‐rotated principal component analyses (VPCAs) 2 to 4, (b) model 2: quadratic fit to S3A OLCI VPCAs 2 to 4, and (c) model 3: linear fit to S3A OLCI VPCAs 1 to 4.

To demonstrate that the VPCA spectral decomposition method is effective at partitioning the contribution of different optical constituents in the IRL, we generated three Chl a estimation models (Figure 9) using Sentinel‐3A OLCI VPCA retrievals and compared them against published Chl a predictions based on Red‐NIR Chl a algorithms employed in the IRL during the 2011 super bloom (Kamerosky et al., 2015). Comparison of our results against the 2011 super bloom is appropriate because while the super bloom covered a greater extent of the IRL, the concentration of the bloom as characterized by the field samples used to calibrate the algorithms was comparable at 120 to 100 Chl a μg/L (this study and Kamerosky et al., 2015, respectively). Sentinel‐3A OLCI VPCA Chl a Model 1 (Figure 9a) produced an *R* = 0.96 and thus *R* ^2^ = 0.92, which explained a factor of ~2× more variance than the published Red‐NIR algorithms (Table 3). Likewise, at 13.38 ug/L, the RMSE of VPCA Chl a Model 1 was a factor of ~3× smaller than the RMSEs of the Red‐NIR models. The RMSE for Model 1, our preferred satellite derived Chl a algorithm (13.38 ug/L) is only slightly larger than the RMSE of the field‐validation for the Kilroy Chl a fluorometer (12.82 ug/L), demonstrating that the VPCA method removed most of the unrelated signal and extraneous noise.

**Table 3:** Comparison to Kamerosky et al., Remote Sensing, vol. 7, 1441–1460, 2015 Calibration Results

Statistic	Moses et al. (2009)	NDCI Quadratic Mishra and Mishra (2012)	This study (weighted S3A OLCI VPCAs 2 to 4, linear fit) Model 1	This study (weighted S3A OLCI VPCAs 2 to 4, quadratic fit) Model 2	This study (weighted S3A OLCI VPCAs 1 to 4, linear fit) Model 3
*R*	0.66	0.71	0.96	0.97	0.83
*R* ^2^	0.43	0.51	0.92	0.94	0.69
RMSE (ug/L)	37.42	34.93	13.38	11.47	39.9
Std. Err (ug/L)	5.29	4.94	4.46	3.82	13.31
*n*	50	50	9	9	9

Abbreviations: NDCI, Normalized Chlorophyll Difference Index; OLCI, Ocean and Land Colour Instrument; RMSE, oot‐mean‐square error; VPCA, varimax‐rotated principal component analysis.

The Sentinel‐3A OLCI VPCA Chl a Model 2 (Figure 9b) is presented for comparison purposes with the quadratic fit to the NDCI that was reported in Kamerosky et al. (2015). While the *R* ^2^ and RMSE statistics for Model 2 appear better than those for Model 1, closer inspection reveals several flaws with this forced regression. The combination of the intercept of −6.62 μg/L and the model RMSE of 11.47 μg/L can result in negative Chl a estimates for low values of Chl a. But of greater concern, the quadratic term in this forced regression is not statistically significant (*p* = 0.29) due to its high correlation with the linear term. It would not have been included if a stepwise selection criteria were employed due to the inherent multicollinearity. Attempting to use Model 2 on data sets other than the calibration set would likely result in poor performance. The weaker performance (*R* = 0.83, *R* ^2^ = 0.69; RMSE = 39.9 μg/L) of Sentinel‐3A OLCI VPCA Chl a Model 3 (Figure 9c), which include all the signal variance in the four components extracted by the VPCA, is similar to the statistics obtained by the published Red‐NIR algorithms. In fact, the reported *R* ^2^ = 0.69 likely overestimates the *R* ^2^ value that would be obtained from a regression based on *R*_rs_ by ~0.07 (i.e., *R* ^2^ ~ 0.62) because the four component model has already excluded 7% uncorrelated noise during the VPCA extraction. Thus Model 3, which is presented for comparison purposes only, documents that the enhanced performance of VPCA Chl a Model 1 arises from the exclusion of the extraneous suspended sediment variance associated with VPCA 1 (Pattern A).

It is common in remote sensing applications to attribute poor Chl a estimation to inadequate atmospheric correction (e.g., Kudela et al., 2015) or the use of fluorometric Chl a data (e.g., Roesler et al., 2017). While these factors are clearly important and well documented, and HPLC pigment estimates have been shown to be more reliable than fluorometric observations, our IRL results document that signal interference arising from neglecting to remove multicollinearity by spectral unmixing can result in significant degradation in Chl a predictive capability, a result previously documented in studies from the optically complex waters of Lake Erie (Ali & Ortiz, 2016; Ortiz et al., 2013), but which is often overlooked in algorithm development.

The VPCA spectral unmixing method also performed well when compared with Chl a estimation methods from other optically complex water bodies. Kudela et al. (2015) studied the impact of cyanobacterial HABs in Pinto Lake, CA. They report *R* ^2^ = 0.24 for the operational NOAA CI and *R* ^2^ = 0.54 for the Scattering Line Height method applied to HICO data. The VPCA spectral decomposition method also performed as well as or better against several semianalytical Chl a algorithms, which report *R* ^2^ ranging from 0.4 to 0.9, when applied to various sensors in a variety of coastal and inland waters ranging from Case I to optically complex environments (Betancur‐Turizo et al., 2018; Lacava et al., 2018; Pyo et al., 2017; Watanabe et al., 2018).

The VPCA spectral decomposition method presented here has several important differences from the manner in which PCA or Empirical Orthogonal Function (EOF) analysis is generally performed in remote sensing (Laliberté et al., 2018; Qi et al., 2014; Soja‐Woźniak et al., 2017). Traditional PCA outperforms standard band ratio algorithms, and provides robust models, but results in many empirical modes that cannot be directly compared to individual optical constituents. Traditional PCA has generally been applied directly to *R*_rs_ or normalized *R*_rs_ in comparison with log transformed Chl a. In addition, the spectral decomposition is generally based on analysis of the covariance matrix, which is dominated by the largest variance–covariance terms. As a result, variance drops off rapidly with increasing component rank, and a large number of components are needed to capture the majority of the variance in the *R*_rs_ data set.

The VPCA spectral decomposition method differs from traditional PCA or EOF analysis by operating on *derivative transformed R*_rs_ from *all bands* in the visible, which removes scattering effects. The method is also based on spectral decomposition of the *correlation matrix*, not the covariance matrix, which enables extraction of smaller signals, boosting the SNR and detection limit by up to a factor of 20× (Ortiz et al., 2019). The lower detection limit means that the method can be used to detect HABs at much lower concentration, earlier in the season providing more time for advanced public health warnings. The final and perhaps most important step is an orthogonal, varimax‐rotation of the principal component loadings (Kaiser, 1958), which maximizes the separation of variance from individual bands within components, leading to greater specificity and more easily interpreted signals (Ortiz et al., 2017; Ortiz et al., 2019). Fewer varimax‐rotated components are needed to capture the majority of the absorption‐related image variance which remains after the derivative transformation and correlation matrix decomposition. As such, the VPCA transformation is in some ways analogous to an AOP to IOP decomposition, explaining why the method performs similarly well at Chl a prediction in relation to IOPs. These preprocessing and postprocessing differences from PCA allows the VPCA method to extract components that can be matched to identifiable optical constituents using principal component regression. This enables the generation of robust, low rank principal component regressions that explain larger fractions of signal variance with less noise and provide for greater specificity through analysis of the individual modes.

Another advantage of the VPCA method over traditional remote sensing applications that use *R*_rs_ rather than the *R*_rs_ derivative is that the VPCA method is less sensitive to atmospheric errors (as can be seen from Figure 3) and which was documented in Ortiz et al., 2017. The method can thus be employed using operational atmospheric corrections applied to standard Level 2 products. The robust results obtained here cannot be ascribed to chance fits arising from errors in atmospheric correction, the use of cell counts, or fluorometric Chl a data, each of which would produce uncorrelated errors across different instruments that would result in poor predictive performance, not the variety of internally consistent, statistically significant relationships that explain the vast majority of the variance with low error in the field and remote sensing matchups observed here. These advantages also apply geographically, enabling more robust solutions in optically complex waters that would previously confound results. These advantages in temporal and spatial HAB prediction have potential for profound improvement in HAB prediction with positive public health implications.

### 5.2 Brown Tide in the IRL

The highest Ochrophyta cell density and biovolume counts were concentrated in the Banana River region of the IRL where several outdated sewage treatment plants generate combined sewer overflows during heavy rains and much of the nutrient pollution from septic runoff in the IRL occurs (Barile, 2018). According to the Chl a and BGA‐PE pigment concentrations measured at the Sykes Creek Kilroy station, during the period in which the high counts of Ochrophyta were measured in the Banana River, seasonal Chl a and BGA‐PE pigment concentrations are high. From Secchi depth recordings on June 29–30, 2018, the lowest visibility was concentrated in the areas of the Banana River, where OLCI VPC 2 (Pattern B) has a high positive correlation with the YSI probe measurements (Table 2) for Chl a, BGA‐PE, and pH but a negative correlation with specific conductivity and salinity. The Chl a and BGE‐PE measurements, which integrate over the entire algal and cyanobacterial communities were positively correlated with Pattern B (OLCI VPCA −2) and Pattern D (OLCI VPCA −4), two components identified with pigments based on their spectral shapes, indicating that the VPCA had partitioned the algal and cyanobacterial communities. The leading component was positively correlated with the PE:Chl a ratio, indicating that blue‐green algae dominated in parts of the IRL with the higher suspended sediment concentrations. Because there was no statistically significant correlation between Pattern A (OLCI VPCA 1) and either water depth or the ratio of Secchi depth to water depth, we conclude that Pattern A (OLCI VPCA 1) likely represents suspended sediment, rather than bottom reflection. These results indicate that the *A*. *lagunensis* detection during field operations occurred during a well‐developed stage of the brown tide, which was inversely associated with cyanobacteria abundance. Our results in the Banana River agree with previous field studies that observe high seasonal biovolume counts of phytoplankton in this region during warmer temperatures and greater rainfall (Chamberlain & Hayward, 1996; Phlips et al., 2011).

From the Secchi depth and turbidity measurements, it appears that the visibility was lower in the Banana River as opposed to the northern IRL (Table S1). Ecologically, it is plausible for an alga such as *A*. *lagunensis* to occur within a turbid environment due to self‐shading or suspended sediment turbidity because it does not require as much sunlight as other algae (Liu et al., 2001). Aside from *A*. *lagunensis*'s adaptation to lower‐light conditions compared to many cyanobacteria, the limiting nutrient necessary to facilitate its growth is nitrogen in the form of ammonium (Backer et al., 2003; Liu et al., 2001; Liu & Buskey, 2000). There have been well‐documented cases of ammonium release from OSDS as the likely source of the nitrogen that drives *A*. *lagunensis* blooms (Lapointe et al., 2015, 2017). Historically, *A*. *lagunensis* occurs in the Banana River where most septic waste is treated by residential OSDS (Barile, 2018). The presence of a highly permeable, sandy subsurface throughout the Florida coastline could harbor an environment that enables frequent ammonium leeching into to the IRL.

Our results validate previous findings from past field studies through our remote sensing methods, indicating that *A*. *lagunensis* blooms occur in the Banana River. Legacy nutrient pollution from growing agricultural and other human inputs could also provide a viable source of nitrogen to fuel the brown tide (Dunne et al., 2011; Reddy et al., 2011; Yang et al., 2013). While the Secchi depth and turbidity variables are positively correlated with Pattern B (OLCI VPC 2) scores, without field measurements for suspended sediment, N and P, we cannot determine whether *A*. *lagunensis* blooms are also potentially linked to resuspension of legacy nutrients within the IRL. This study indicates that moderate resolution imaging systems can be used to locate and source nutrient polluted areas in large bodies of water. The excellent comparison between the ASD HH2 (~50 cm) and OLCI (300 m) data despite differences in spatial resolution also indicates that the method employed here is independent of the spatial scale of the results, which opens up tremendous opportunities. Stakeholders can compare results between handheld, UAV, aerial, or orbital sensors to make integrative policy decisions. The use of handheld sensors or sensor‐equipped drones with small spot size allows application to lakes and ponds smaller than can be studied by orbital remote sensing. The ability for data fusion also has implications for inter‐comparison of results from proposed orbital hyperspectral missions such as the Plankton, Aerosol, Cloud, ocean Ecosystem (PACE) mission or the Surface Biology and Geology (SBG) mission concept as recommended in the NASA decadal survey (NASEM, 2018).

## 6 Conclusions

The results presented here document that the VPCA method can be calibrated by spectral principal component regression against a known library and validated by spatial principal component regression against independent field observations of cell density, cell biovolume, or Chl a measurements. By using the KSU VPCA method to partition the integrated optical water signal into independent components, rather than only employing traditional Chl a algorithms, we separate out spectral signatures that have similar reflectance derivative peaks and troughs, and better elucidate the location and composition of the brown tide to determine how it varies with time. By recombining the information in the components that exhibit a red‐edge response, it is possible to generate a Chl a algorithm that outperforms empirical band ratio algorithms and preforms as well or better than a variety of semianalytical algorithms. The results from the VPCA spectral decomposition method are more efficient than traditional EOF or PCA methods, requiring fewer components in algorithms that explain as much or more variance. The components extracted by VPCA are also diagnostic for identifiable optical constituents, providing greater specificity in the resulting data products.

These advances are significant with respect to remote sensing applications, indicating greater potential to generalize the results, and the potential for much greater specificity in data products derived from future hyperspectral missions, such as the NASA PACE mission or the SBG mission concept.

The information extracted by VPCA is also important from a public health standpoint. Given correlations of the *A*. *lagunensis*‐related OLCI VPCA component loadings to Ochrophyta cell counts and relatively high suspended sediment in brackish conditions, our results indicate an environment suitable for abundant brown tide blooms (Liu & Buskey, 2000). While we hypothesize that the primary driver for brown tides could be from septic runoff, our analyses do not provide enough evidence to rule out other potential drivers such as fertilizer runoff or legacy nutrient sources. Future field sampling should require tests for fecal coliform and elemental analyses of water samples for N and P to discriminate between the multiple, potential sources. Regardless of which nutrient source within the IRL is dominant, we can conclude that the distribution of the *A*. *lagunensis*‐related spectral components are abundant in the region of the Banana River, along with multiple, strong correlations between different remote sensing instrumentation and cell count information. The response to Chl a and cell counts were linear from a detection limit close to zero up to Chl a values on the order of 120 ug/L and for Ochrophyta cell biovolumes up to 10^10^ μm^3^/L. By quantifying the minimum Chl a and cell count biovolume concentration necessary for brown tide detection, we demonstrate that the VPCA method can be used to reliably monitor the brown tide from very low levels to full bloom concentrations. This research has shown that when spectral signatures of organisms representative of a region of interest are added to the spectral library, the VPCA components can be better characterized to known constituents and correlated to field cell count measurements. This provides a viable method to monitor specific types of HABs in optically complex environments to aid stakeholders in developing public policy and to safeguard public health.

## Conflict of Interest

The authors declare no conflicts of interest relevant to this study.

## Supporting information

Supporting Information S1
