# Supplementary material for: Field‐Validated Detection of Aureoumbra lagunensis Brown Tide Blooms in the Indian River Lagoon, Florida, Using Sentinel‐3A OLCI and Ground‐Based Hyperspectral Spectroradiometers
Source: Geohealth. 2020 Jun 20;4(6):e2019GH000238. doi: 10.1029/2019GH000238 (PMC7305661; doi:10.1029/2019GH000238)
Supplement: Supplementary file 1 — Supporting Information S1 [file GH2-4-e2019GH000238-s001.docx]

**Supplemental Information: Field-validated detection of *Aureoumbra lagunensis* brown tide blooms in the Indian River Lagoon, Florida using Sentinel-3A OLCI and ground-based hyperspectral spectroradiometers**

**Taylor J. Judice^1^, Edith A. Widder^2^, Warren H. Falls^2^, Dulcinea M. Avouris^1^, Dominic J. Cristiano^1^, Joseph D. Ortiz^1^**

^1^Kent State University Department of Geology, Kent OH, USA, ^2^Ocean Research and Conservation Association, 1420 Seaway Drive, Fort Pierce, FL, USA

Corresponding author: Joseph D. Ortiz ([jortiz@kent.edu](mailto:jortiz@kent.edu))

Full Materials and Methods

*1. Continuous autonomous field sampling*

We conducted remote sensing analysis of Sentinel-3A OLCI images that covered the Northern Indian River Lagoon over an ~1.5-year time span (7/31/17 to 1/1/19) to isolate the signature of the brown tide that develops there (Table 1). For field comparison with our remote sensing results, we employed several types of measurements, including continuous water-quality data, obtained using the Ocean Research and Conservation Association’s (ORCA) piling-mounted water-quality measuring Kilroy^TM^ instrumentation (Thosteson et al., 2009). A total of five (5) fixed stations housed automated Kilroy^TM^ environmental sensing systems that were operated and maintained by ORCA during the course of the study (Figure 1b, Supplemental Figure S1a-b). These devices record real time water-quality parameters including Chl a and Blue-Green Algae phycoerythrin (BGA-PE) as a measure of blue-green algae continuously in ~30-minute increments year-round with occasional interruptions for regular maintenance. The observations are telemetered back to the lab and reported via the internet for public viewing using the Kilroy livefeed app (<http://api.kilroydata.org/public/>) which is accessed from the ORCA website (<https://www.teamorca.org>). Archived data is accessible via a user customizable plotting interface.

The time series of fluorometrically measured pigments available from the Kilroy^TM^ systems are located at: Turnbull Creek, Haulover Canal, Barge Canal, Sykes Creek, and Dragon Point. Sykes Creek is the only Kilroy^TM^ sensor that was located within the Banana River. The Kilroy^TM^ instruments are calibrated against rhodamine dye for each parameter using a two-point calibration in the lab following manufacturer specifications before deployment. For Chl a, the measurement range is from 0 to 400 ug/L. For the BGA-PE probe the measurement range is from 0 to 280 ug/L. The remote, real time data is reviewed daily to assess environmental changes and potential sensor drift by techs at ORCA. The instruments are physically checked in the field against a calibrated Yellow Springs Instruments^TM^ (YSI) EXO2 Multiparameter sonde during site visits by ORCA techs at least monthly. Instruments are recalibrated as needed, but not less frequently than every 6 months if no drift is observed. Instruments that are not preforming to specifications during the routine physical inspections are replaced immediately if a calibrated spare is available or removed for service and re-calibration in the lab before re-deployment. A field-based validation of the Barge Canal Kilroy Chl a and BGA-PE probes based on 11-months of field check data over a 12-month period yields R=0.95, R^2^ = 0.91, and RMSE = 12.82 ug/L with *p* = 6.2 x 10^-6^ for Chl a, and R=0.93, R^2^ = 0.87 and RMSE = 23.95 ug/L with *p* = 2.0 x 10^-5^ for BGA-PE, documenting the stability of the calibrations in the field.

*2. Field Campaign for Remote Sensing Calibration*

For image calibration, we implemented field sampling in the IRL on June 29-30, 2018 during a peak in the brown tide. Sampling was conducted from small boats with the assistance of collaborators from ORCA. Measurements were collected from 11 locations in the IRL: six samples from the northern Indian River Lagoon and five samples from the Banana River (Figure 1b; Supplemental Table S1). The field work was conducted at each of the Kilroy sites and additional locations selected to provide geographic coverage for comparison with the image acquired on June 28, 2018. Nine sites were cloud-free for comparison with remote sensing pixels. To ensure spatial consistency for comparison with Sentinel-3A OLCI imagery, GPS coordinates were recorded at all locations for each sample collection and the closest OLCI pixel was selected for comparison. At each station, we measured hyperspectral surface reflectance using a handheld spectroradiometer and hydrographic parameters with a YSI EXO2 Multiparameter sonde (including Chl a and BGA-PE), and collected water samples for single blind, cell count density (cells/L) and biovolume (μm^3^/L) measurements by an independent, commercial lab (BSA Environmental Services, Inc., Beachwood, OH), along with suspended sediment samples, and Secchi depth measurements. We compare against cell counts because they are the metric employed by water quality managers (e.g. WHO, OEPA, USACE-Pittsburgh) to assess HAB conditions and take policy action (WHO, 1999; Wynne et al. 2010; Davis et al., 2019; OEPA, 2019; USACE-Pittsburgh, 2020). Cell counts also provide algal and cyanobacterial community abundance information useful to assess the reliability of the spectral decomposition method employed here. These field samples provided *in-situ* data for calibration of the remote sensing analysis.

During field surveys, a YSI EXO2 Multiparameter sonde was used at all locations to record profiles of Chl a, BGA-PE, pH, salinity and turbidity. For cell count analyses, two replicate 125 mL surface water samples were collected at each of the 11 locations. Once obtained, the cell count samples were preserved using 2 mL of 5% Lugols solution per 25 mL of sample. Bottom sediment samples (assumed to be representative of the type of suspended sediment found at each location) were collected with a clamshell sampler to increase the amount of usable sample for Scanning Electron Microscope (SEM-EDS) analyses, then stored in clear 125 mL containers. All water and wet sediment samples were kept cold on ice until returning to the lab for processing and shipment.

Using an Analytical Spectral Devices^TM^ (ASD) FieldSpec^®^ Handheld 2 (HH2) hyperspectral spectroradiometer, we measured the absolute surface water reflectance at the 11 sample locations using a 10-degree field of view foreoptic attachment, yielding a pixel size of ~50 cm given the elevation of the instrument above the water surface. Measurements were collected to avoid sun glint, but the absolute measurement geometry is less important in our application that traditional remote sensing methods because the KSU spectral decomposition method relies on derivative spectroscopy, rather than direct analysis of reflectance spectra which minimizes geometric interferences (Ortiz et al., 2017). The reflectance spectra from the hyperspectral ASD FieldSpec^®^ HH2 were averaged to 10 nm resolution from 400-700 nm. This provides a more continuous 31 bands compared to the 11 multispectral bands of the Sentinel-3A OLCI sensor. The hyperspectral data were also resampled to the Sentinel-3A OLCI band resolution for direct comparison.

The hyperspectral field instrument allows us to obtain surface water reflectance with much less atmospheric interference and much higher sample counts than retrievals from satellite imagery to minimize noise. For each FieldSpec^®^ HH2 surface reflectance measurement the optics were calibrated with an ASD standard white reference Spectralon^TM^ plate before orienting the instrument over the water for nadir-viewing under full ambient illumination using a ~1.5 m long boom to reduce adjacency effects while obtaining surface reflectance measurements. To minimize noise and increase the signal-to-noise ratio (SNR) of the surface water reflectance measurements, each instrument measurement was averaged in several steps. Each individual spectrum was collected as an integration of 30 spectra within the instrument software. These 30-spectra integrations were then set to auto-collect in groups of eight. At each site, we collected 5 to 10 groups of 8 spectra each depending on available time and the stability of the measurement, thus yielding a grand sample average for each site based on 1200 to 2400 individual spectra. Spectra that were saturated or outliers due to poor measurement geometry (e.g. off nadir pointing) were discarded and not included in the grand average. The site-averaged reflectance values were then transformed to centered-weighted, first derivatives following Press et al., (1992) to remove the low-frequency part of the signal, which is dominated by backscatter in preparation for VPCA.

*3. Lab analysis of field data*

Cell count samples were kept refrigerated until shipped to BSA Environmental Services, Inc. (Beechwood, Ohio) for analysis. Each sample was identified to the genus and species level, measuring both cell density (cells/L) and cell volume (μm^3^/L). To compare reflectance data with the cell count results, samples were integrated to the Phylum (Division) level to account for ambiguity in estimation by remote sensing because the optical signal integrates over the entire plankton community. Results were averaged between the two replicate samples at each location.

To validate the identification for the other spectral components, which were related to minerals, such as clays, hematite or goethite, SEM-EDS was used to determine the presence of clays and iron minerals within the dried sediment samples. The sediment collected with the clamshell sampler was wet sieved to grain sizes <63 μm to simulate suspended material that could be observed in the integrated *R_rs_* spectral signature from Sentinel-3A OLCI imagery. After sieving, sediment was dried in an oven at 60°C, hand ground with a mortar and pestle to homogenize the signal, and adhered to aluminum tacks for SEM-EDS analysis. This analysis was aimed at providing a qualitative assessment of presence/absence of clays or iron-bearing minerals, and thus mineral stoichiometry from the SEM-EDS was not conducted.

Sediment analyses by SEM-EDS provide useful information on the elemental chemistry and a qualitative assessment of the minerals present in the IRL through visual inspection. From the SEM-EDS analyses, the sediment samples contain clay minerals and some type of iron-bearing minerals or iron-coated sediment grains. In all sediment samples the bulk mineral composition contained primarily clay minerals with the inclusion of modern marine shell fragments and sand-sized grains of quartz with clear evidence of conchoidal fracturing. From bulk elemental composition, all sediment samples contained minor grains of primarily iron-bearing material. While no definite mineral crystal structure could be observed in the iron grains, the size of these iron-bearing grains range on average between ~10 μm and ~30 μm. The confirmation of clay minerals and iron presence in our field samples provides sufficient validation for the type of mineral constituents identified in the OLCI VPCA decomposition, although there is ambiguity from our SEM measurement as to whether these iron grains were hematite, goethite, or some other iron-bearing mineral on the basis of the backscatter data alone. In addition, these results are consistent with previous surveys of the IRL, which have found iron in water samples (Trocine and Trefry, 1996).

*4. Lab culture reflectance derivative spectrum analysis*

In addition to collecting water samples for cell counts, we measured the reflectance of a filtered *Aureoumbra lagunensis* culture provided by ORCA laboratories, as an example spectral response for members of the Ochrophyta. *A. lagunensis* was cultured in a 500 mL flask under standard temperature control and sterile conditions. From the 500 mL of culture, 250 mL of the *A. lagunensis* sample was filtered through 47 mm diameter glass microfiber filters (GF/F) with 0.7 μm pore size at <15 psi to prevent cell breakage (lysing), which minimizes the distortion to pigment packaging that can occur when pigments are extracted by solvents (Bricaud et al., 2004; Lohrenz et al., 2003). These filters accumulate the particulate material within the water, but do not capture any colored dissolved organic matter. Following procedures similar to Ortiz et al., (2013), the two filtered water samples were dried at 60° C for ~1 hour to remove water. Once the filtered samples dried, they were measured using an ASD FieldSpec^®^ HH2 equipped with a High-Intensity contact probe that utilizes a light source of known optical properties. The device was calibrated against a white Spectralon^TM^ plate (9 cm diameter) then the sample was placed on a scissor jack and raised up to the device until sealed beneath the contact probe. With the filter in contact with the probe, ambient light was excluded from any measurements. Approximately 1200 individual reflectance values were measured for the particulates accumulated on the GF/Fs as well as for unfiltered blank reference GF/Fs. The sample reflectance spectra were averaged and blank-corrected for the absorption from the white GF/F background by division of the reflectance sample average using the averaged blank filter measurement. As with the field reflectance measurements, all GF/F reflectance values were averaged to one reflectance spectra for each site on each day and then transformed to centered derivatives, which removes scattering affects. The spectrum of the culture sample is presented at both 10 nm hyperspectral resolution and Sentinel-3A OLCI resolution for comparison with the field collected spectra from the ASD and orbital sensor. The *A. lagunensis* spectrum was included in a library of known spectral constituents (Ortiz et al., 2017; Ortiz et al., 2019), which was used for later component loading identification following the VPCA decomposition.

*5. Remote Sensing Image Analysis and VPCA Spectral Decomposition*

The Kent State University (KSU), varimax-rotated, principal component analysis (VPCA) method, a level-3 remote sensing transformation, was developed for decomposing the integrated, spectral signature from optically complex water retrieved by remote sensing instruments. The method partitions reflectance derivative spectra into independent component spectral signatures, which account for quantifiable percentages of the total image variance (Avouris and Ortiz, 2019; Ortiz et al., 2013; Ortiz et al., 2017, Ortiz et al., 2019). The method is relatively insensitive to atmospheric error (Ortiz et al., 2017) and can be applied to level-1 data that are then atmospherically corrected, or to standard level-2 atmospheric corrected data products. In fact, VPCA improves on traditional atmospheric correction by partitioning signal from noise (Ortiz et al., 2017). In this application, the starting point for the analysis was the standard EUMETSAT level-2 atmospheric corrected data product for Sentinel-3A. The atmospheric correction was the ATBD Atmospheric Corrections Bright Water Correction protocol provided by EUMETSAT, which is an updated version of the atmospheric correction algorithm developed for MERIS (Sentinel-3 OLCI Marine User Handbook, 2018; Moore and Lavender, 2010).

The VPCA method addresses the mixed pixel problem arising from the presence of multiple constituents in optically complex water by partitioning their signals into independent, uncorrelated components (Ortiz et al., 2019). In this way, the method separates signatures of bottom reflection, suspended sediment and pigment degradation products from various types of algal and cyanobacterial pigments because different processes or targets yield distinct spectral responses (Supplemental Table S2). The extracted components represent mixtures of constituents that can be characterized by forward, stepwise, principal component regression against a library of known algae, cyanobacteria, pigments, and sediments (Avouris and Ortiz, 2019; Ortiz et al., 2013; Ortiz et al., 2017, Ortiz et al., 2019). The method was initially developed for HAB detection in Lake Erie (Ortiz et al., 2013) and is described in detail elsewhere (Ortiz et al., 2019) and validated by several studies (Avouris and Ortiz, 2019; Ortiz et al., 2013). By partitioning spectral signatures into orthogonal (independent) components and identifying them, derived products from satellite images faithfully account for constituents in the water without bias from intercorrelated variables.

We obtained and evaluated Sentinel-3A OLCI level-2 image products with 300 x 300 m water pixels in the IRL study area from the EUMETSAT Copernicus data repository. A total of 12 images between 7/31/17 and 1/1/19 with minimal cloud cover at the Sykes Creek Kilroy location (Figure 1b), located within the Banana River region of the IRL were selected for analysis (Table 1). The June 28, 2018 image acquired one to two days before the two-day field sampling work on 6/29/18 to 6/30/18 was used for comparison with field observations due to its near coincident timing and minimal cloud coverage. Using Harris Geospatial ENVI/IDL software, the center-weighted derivative for the 11-visible bands in the Sentinel-3A OLCI spectra were calculated for the data from each image following the numerical method presented in Press et al., (1992).

To isolate the signal associated with the brown tide bloom, we conducted separate VPCA, on the ASD Fieldspec^®^ HH2 field data and the Sentinel-3A OLCI derivative spectra datasets obtained from each of the 12 images. VPCA reduces the dimensionality of multivariate datasets by decomposing the integrated spectral signatures into orthogonal axes that each account for a portion of the total signal variability (Ortiz et al., 2019). In this way, it removes redundant information from the image. The derivative spectra from the images and field samples were analyzed with a forward orthogonal PCA rotation, which computes the eigenvalues and eigenvectors. This result was then further transformed using an orthogonal, varimax rotation (Kaiser, 1958), providing better separation of the component loading coefficients while preserving the orthogonality of the eigenvectors. This improves the interpretability of the resulting components (Kaiser, 1958). Because the orientation of the varimax rotation is arbitrary, the axes can be flipped based on comparison with field data to constrain the sign of the component. The eigenvectors describe the axes of the new system, while the eigenvalues describe how long each axis is, thus providing a measure of the percent variance described. The number of spectral components to extract from the VPCA was set to the number of eigenvalues greater than 1, plus one additional component. Following the varimax rotation, the results yield component loadings, which represent independent spectral signatures of constituents in the water column, and component scores, which represent the spatial distribution of these spectral signatures. For each day, the components are sorted in variance rank order, but the variance explained by each component can vary with time. As a result, component rank is not diagnostic of composition. For this reason, we match the numerically ranked components extracted from our calibration image (6/28/18) with the components for the remaining days in the data set into component groups designated as Pattern A, B, C, and D, which correspond to the repeating, independent spectral signals extracted from the images. For satellite images, the component scores were displayed as distribution maps, which represent the fractional variance associated with each component at each pixel, while the component scores of the field spectra represent single location values at the field sampling site. These orthogonal VPCA component loadings and scores were calculated using code employed in ENVI/IDL by (Avouris and Ortiz, 2019; Ortiz et al., 2017, Ortiz et al., 2019) for multispectral satellite imagery and in SPSS for hyperspectral spectroradiometer data.

*6. Validation and Spectral Identification*

The VPCA component loadings obtained for all datasets were identified by forward stepwise principal component regression against a spectral constituent library (Avouris and Ortiz, 2019; Ortiz et al., 2019), with the inclusion of the additional spectra measured from the filtered *A. lagunensis* culture. We also correlate the extracted VPCA spectra directly with the reference spectrum for *A. lagunensis* measured from the culture. The library of known reflectance derivative spectra for water quality constituents includes 84 signatures: 10 for algal groups or taxa, 27 extracted algal and cyanobacterial pigments and accessory pigments, 6 chlorophyll a and b degradation products, and 41 minerals compiled from the literature (Ortiz et al., 2013, 2019 and references therein), using mineral spectra from the United States Geological Survey (USGS) Spectral Library (Kokaly et al., 2017) or measured in the lab (Ortiz et al., 2013, 2019).

Use of principal component regression addresses the main weakness of stepwise multiple linear regression and provides robust results because the signals extracted from the images are independent, minimizing the potential for multicollinearity in the results. In addition to standard regression statistics (R, R^2^, *F*-value, *p*-value), the level of multicollinearity on a term by term basis was assessed using the variance inflation factor (VIF). The individual VPCA loadings were fit to as many matching spectral constituents in the library as needed before reaching a stopping criterion of VIF ≤ 2 for all terms in the equation, rather than the customary value of 4-5. This approach minimizes the risk of over fitting (Ortiz et al., 2019). The VIF values are reported as the range of calculated values when different, or as a single value if identical VIFs were found for all terms. Even with a VIF threshold as low as 2, statistically significant fits that explain the majority of variance in the VPCA components (R^2^ ≥ 0.87) were obtained with mixtures of only two to three constituents due to the orthogonal nature of the principal components. Once the spectral signatures of all component loadings were identified using the constituent library, the component loadings or scores for the two instrument datasets were correlated against each other. Correlations between the two datasets was useful to demonstrate consistency of the KSU VPCA spectral decomposition method across the hyper- and multispectral instruments. To further validate spectral identifications with *A. lagunensis* and other potential constituents, we correlated the spatially-related, component scores from the field instrument and extracted component score pixel values from the VPCA distribution map of the Sentinel-3A image to the phylum-level cell density and biovolume results obtained for each sample site and the YSI EXO2 sonde parameters.

This approach addresses the mixed pixel problem for optically complex water by separating out image variance due to extraneous factors such as bottom reflectance, suspended sediment, CDOM, or other types of algae and cyanobacteria or random noise from the signature of interest, in this case the spectral response of *A. lagunensis* or related species. We present the spectral shapes and identification for four components extracted from our validation image, but focus on the signature of the brown tide related component for the remaining images because the focus of this applied geohealth-related paper is assessment of the HAB by remote sensing. The other extracted VPCA components could, however, be used to provide information regarding the distribution of suspended sediment, the distribution of pigment degradation products or to assess changes in algal community structure with space and time. Topics that can be discussed in future publications.

*7. Comparison with prior studies: Chl a estimation*

Prior remote sensing studies of the Indian River Lagoon reconstructed bloom intensity and extent using Chl a as a proxy for biomass. Kamerosky et al. (2015) applied the red edge band ratio algorithms of Moses et al., (2009) and Mishra and Mishra (2012) to estimate Chl a in the IRL during the 2011 super bloom. The algorithm of Mishra and Mishra (2012) is referred to as the Normalized Chlorophyll Difference Index (NDCI). The algorithms are two-band Red-NIR algorithms that make use of a band centered on the red edge to monitor Chl a, which is normalized against an NIR band assumed to have minimal interference from Chl a. These algorithms were originally devised for Chl a estimation in the Sea of Azov (Moses et al., 2009) and Chesapeake, Delaware, and Mobile Bays and the Mississippi Delta (Mishra and Mishra (2012), all of which are optically complex estuarine or marginal marine environments prone to harmful algal blooms. Kamerosky et al. (2015) regionally tuned the algorithms for use in the IRL by calibration with a data set of 50 samples, and then validated them using an addition 40 independent samples that were withheld from the calibration analysis. This study compares against their calibration results.

For comparison with Kamerosky et al. (2015), we built three simple principal component regressions from the extracted VPCA components, calibrated with the nine Chl a measurements from the YSI EXO sonde collected at the cloud-free locations of our field samples. The dependent variable for the first model was the weighted sum of the Sentinel-3A VPCA scores, where the weighting factor was the fractional variance explained by each of the three components that exhibited a red-edge response (VPCA 2, 3 and 4 or patterns B, C, D). For this first model, VPCA 1 (Pattern A), which did not exhibit a red-edge response and which was identified as primarily related to the mineral illite was excluded. We compared linear and quadratic fits to the Chl a data for the first and second model for direct comparison with the results from Kamerosky et al. (2015). For comparison purposes and to show the importance of the spectral decomposition, we also constructed a third model, which was based on the variance-weighted sum of VPCA 1 to 4 (patterns A, B, C, and D), which was regressed against the field Chl a data. Model 3 is “cleaner” than a result based on raw reflectance data because the four ASD Field Spec HH2 VPCA components exclude ~7% noise, but including all the components mimics the effect of generating a regression using unmixed reflectance data.

**References**

Avouris, D. M., & Ortiz, J. D. (2019). Validation of 2015 Lake Erie MODIS image spectral decomposition using visible derivative spectroscopy and field campaign data. *J. Great Lakes Res.* 45, 466–479.

Bricaud, A., Claustre, H., Ras J., & Oubelkheir, K., (2004). Natural variability of phytoplanktonic absorption in oceanic waters: Influence of the size structure of algal populations. *J. Geophys. Res., Oceans,* 109(C11).

Davis, T. W., Stumpf, R., Bullerjahn, G. S., McKay, R. M., Chaffin, J. D., Bridgeman, T. B., & Winslow C. (2019). Science meets Policy: A framework for determining impairment designation critewria for large waterbodies affected by cyanobacterial harmful algal blooms. *Harmful Algae,* 81, 59-64.

Kaiser, H. F. (1958). The varimax criterion for analytic rotation in factor analysis. *Psychometrika* 23, 187–200.

Kamerosky, A., Cho, H. J., & Morris, L. (2015). Monitoring of the 2011 super algal bloom in Indian River Lagoon, FL, USA, Using MERIS. *Remote Sens.,* 7, 1441–1460.

Kokaly, R. F., Clark, R. N., Swayze, G. A., Livo, K. E., Hoefen, T. M., Pearson, N. C., et al., (2017). *USGS Spectral Library Version 7*. United States Geological Survey Data Series 1035 doi:10.3133/ds1035.

Lohrenz, S., Weidemann, A. D., & Tuel, M., (2003). Phytoplankton spectral absorption as influenced by community size structure and pigment composition. *J. Plankton Res.* 25(1), 35-61.

Mishra, S., & Mishra, D. R. (2012). Normalized difference chlorophyll index: A novel model for remote estimation of chlorophyll a concentration in turbid productive waters. *Remote Sens. Environ.,* 117, 394–406.

Moore, G., & Lavender, S. (2010). OLCI Bright Waters AC (mesotrophic to high turbidity), Algorithm Theoretical Basis Document, Sentinel-3 L2 Products and algorithm Definitions, Argans and BioOptika, Document Ref: S3-L2-SD-03-C08-ARG- ATBD_BWAC, v2.1, 40 pg.

Moses, W.J., Gitelson, A. A., Berdnikov, S., & Povazhnyy, V. (2009). Estimation of chlorophyll-a concentration in case II waters using MODIS and MERIS data—Successes and challenges. *Environ. Res. Lett.,* 44, doi:10.1088/1748-9326/4/4/045005.

Ohio Environmental Protection Agency (2019). Developing a Harmful Algal Bloom (HAB) Treatment Optimization Protocol - Guidance for Public Water Systems. Division of Drinking and Ground Waters, Columbus, Ohio, Version 2.1 January 2019, 33pp.

Ortiz, J. D. Avouris, D., Schiller, S., Luvall, J., Lekki, J., Tokars, R. P., et al. (2017). Intercomparison of Approaches to the Empirical Line Method for Vicarious Hyperspectral Reflectance Calibration. *Front. Mar. Sci.,* 4, <https://doi.org/10.3389/fmars.2017.00296>.

Ortiz, J. D., Avouris, D., Schiller, S. J., Luvall, J. C., Lekki, J. D., Tokars, R. P., et al. (2019) Evaluating visible derivative spectroscopy by varimax-rotated, principal component analysis of aerial hyperspectral images from the western basin of Lake Erie. *J. Great Lakes Res.,* 45(3), 522-535, <https://doi.org/10.1016/j.jglr.2019.03.005>.

Ortiz, J. D., Witter, D. L., Ali, K. A., Fela, N., Duff, M., & Mills, L., (2013). Evaluating multiple color producing agents in Case II waters from Lake Erie. *International Journal of Remote Sensing,* 34 (24), 8854-8880.

Press, W. H., Teukolsky, S. A., Vetterling, W. T., & Flannery, B. P. (1992). Numerical Recipes in Fortran. New York, NY: Cambridge University Press.

Sentinel-3 OLCI Marine User Handbook (2018). Document number: EUM/OPS-SEN3/MAN/17/907205, v1H, EUMETSAT, Eumetsat-Allee 1, D-64295 Darmstadt, Germany, 41 pg.

Thosteson, E. D., Widder, E. A., Cimaglia, C. A., Taylor, J. W., Burns B. C., & Paglen, K. J., (2009). New technology for Ecosystem-Based Management: Marine monitoring with the ORCA Kilroy Network. In OCEANS 2009-EUROPE 1–7 (IEEE, 2009). dDoi:10.1109/OCEANSE.2009.5278229

Trocine, R. P., & Trefry, J. H. (1996). Metal concentrations in sediment, water and clams from the Indian River Lagoon, Florida. *Mar. Pollut. Bull.,* 32, 754–759.

U.S. Army Corps of Engineers Pittsburgh, District Harmful Algae Blooms (HABs), online report, <https://www.fs.usda.gov/Internet/FSE_DOCUMENTS/fseprd518784.pdf>, [accessed: Feb 15, 2020].

World Health Organization, (1999). *Toxic Cyanobacteria in Water: A Guide to Their Public Health Consequences, Monitoring and Management*, (Chorus, I.; Bartram, J., eds.), CRC Press: Boca Raton, FL, USA.

Wynne, T. T., Stumpf, R. P., Tomlinson, M. C., & Dyble, J. (2010). Characterizing a cyanobacterial bloom in western Lake Erie using satellite imagery and meteorological data. *Limnol. Oceanogr*., 55(5) 2025-2036.

**Figure Captions**

**Supplemental Figure S1.** (a) Image of one Kilroy instrument suite (Thosteson et al., 2009), (b) mounted on submerged piers at five locations along the northern IRL.
